# Association between Neu5Gc carbohydrate and serum antibodies against it provides the molecular link to cancer: French NutriNet-Santé study

**DOI:** 10.1186/s12916-020-01721-8

**Published:** 2020-09-23

**Authors:** Salam Bashir, Leopold K. Fezeu, Shani Leviatan Ben-Arye, Sharon Yehuda, Eliran Moshe Reuven, Fabien Szabo de Edelenyi, Imen Fellah-Hebia, Thierry Le Tourneau, Berthe Marie Imbert-Marcille, Emmanuel B. Drouet, Mathilde Touvier, Jean-Christian Roussel, Hai Yu, Xi Chen, Serge Hercberg, Emanuele Cozzi, Jean-Paul Soulillou, Pilar Galan, Vered Padler-Karavani

**Affiliations:** 1 Department of Cell Research and Immunology, The Shmunis School of Biomedicine and Cancer Research, The George S. Wise Faculty of Life Sciences, Tel Aviv University, Tel Aviv, 69978 Israel; 2 Sorbonne Paris Cité Epidemiology and Statistics Research Center (CRESS), Inserm U1153, Inra U1125, Cnam, Paris 13 University, Nutritional Epidemiology Research Team (EREN), Bobigny, France; 3 Department of Thoracic and Cardiovascular Surgery, Institut du Thorax, University Hospital, Nantes, France; 4 Department of Cardiology, Institut du Thorax, University Hospital, Nantes, France; 5 Service de virologie Centre Hospitalo-Universitaire de Nantes, F44093 Nantes, France; 6 Institute of Structural Biology, University Grenoble Alpes, UMR CNRS CEA UGA 5545 CEA, CNRS 38044, F38042 Grenoble, France; 7 Department of Chemistry, University of California-Davis, Davis, CA 95616 USA; 8 Transplant Immunology Unit, Department of Cardiac, Thoracic and Vascular Sciences, Padua University Hospital, Padua, Italy; 9 Centre de Recherche en Transplantation et Immunologie UMR 1064, INSERM, Université de Nantes, Nantes, France

**Keywords:** Red meat, Cancer, Sialic acid, Antibodies

## Abstract

**Background:**

High consumption of red and processed meat is commonly associated with increased cancer risk, particularly colorectal cancer. Antibodies against the red meat-derived carbohydrate *N*-glycolylneuraminic acid (Neu5Gc) exacerbate cancer in “human-like” mice. Human anti-Neu5Gc IgG and red meat are both independently proposed to increase cancer risk, yet how diet affects these antibodies is largely unknown.

**Methods:**

We used world global data to demonstrate that colorectal cancer incidence and mortality are associated with increased national meat consumption. In a well-defined large cohort, we used glycomics to measure daily Neu5Gc intake from red meat and dairy, and investigated serum as well as affinity-purified anti-Neu5Gc antibodies. Based on 24-h dietary records, daily Neu5Gc intake was calculated for 19,621 subjects aged ≥ 18 years of the NutriNet-Santé study. Serum and affinity-purified anti-Neu5Gc antibodies were evaluated by ELISA and glycan microarrays in representative 120 individuals, each with at least eighteen 24-h dietary records (aged 45–60, Q1–Q4; aged > 60, Q1 and Q4; 10 men/women per quartile).

**Results:**

We found that high-Neu5Gc diet, gender, and age affect the specificity, levels, and repertoires of anti-Neu5Gc IgG immune responses, but not their affinity. Men consumed more Neu5Gc than women, mostly from red meat (*p* = 0.0015), and exhibited higher overall serum anti-Neu5Gc IgG levels by ELISA (3.94 ng/μl versus 2.22 ng/μl, respectively; *p* = 0.039). Detailed glycan microarray analysis against 56 different glycans revealed high Neu5Gc-specificity with increased anti-Neu5Gc IgG and altered repertoires, associated with higher consumption of Neu5Gc from red meat and cow dairy. Affinity purification of serum anti-Neu5Gc antibodies revealed increased levels and biased array repertoire patterns, without an increase in antibody affinity, in individuals consuming higher Neu5Gc levels. Furthermore, in a high-meat diet, antibody diversity patterns on glycan microarrays shifted towards Neu5Gcα3-linked glycans, increasing the α3/α6-glycans ratio score.

**Conclusions:**

We found a clear link between the levels and repertoire of serum anti-Neu5Gc IgG and Neu5Gc intake from red meat and dairy. These precise rational methodologies allowed to develop a *Gcemic index* to simplify the assessment of Neu5Gc in foods that could potentially be adapted for dietary recommendations to reduce cancer risk.

## Background

Nutrition can dramatically affect health, and different dietary habits have been associated with various human diseases such as cancer, cardiovascular diseases, type II diabetes, obesity, and hypertension [1–4]. In particular, high consumption of red meat has been frequently suggested as a risk factor for human cancers and cardiovascular diseases [1–4]. Although various mechanistic explanations have been proposed (e.g., high energy/fat diet, N-nitroso, nitrates, nitrites, heme iron, compounds produced by gut microbiome or during cooking), none seems to be specific for red meat or dairy [5]. Recently, based on limited evidence in humans, the non-human carbohydrate *N*-glycolylneuraminic acid (Neu5Gc) that is present in mammalian-derived food (i.e., red meat and dairy) has been implicated as a new risk factor for colorectal cancer [2].

Neu5Gc is a common sialic acid type of sugar in mammals. It is a nine-carbon negatively charged monosaccharide that can be synthesized by most mammals and found at the tips of carbohydrate chains (glycans), glycoproteins, and glycolipids [6]. Humans cannot synthesize Neu5Gc due to a deletion in the *CMAH* gene that encodes the cytidine 5′-monophosphate-Neu5Ac hydroxylase [7]. Yet, dietary Neu5Gc can be consumed then incorporated at low levels onto human cell surfaces, particularly in cancer, consequently displaying a broad assortment of immunogenic Neu5Gc-glycans [6, 8]. In fact, all humans examined thus far have a diverse collection of polyclonal anti-Neu5Gc antibodies [7, 9, 10]. Thus, circulating anti-Neu5Gc antibodies continuously encounter Neu5Gc-containing epitopes on human tissues and have been proposed to lead to xenosialitis [11], which in mice have been shown to exacerbate cancer [11, 12] and cardiovascular disease [13]. Diverse feeding methods in human-like Neu5Gc-deficient *Cmah*^−/−^ mice failed to recapitulate diet induction of anti-Neu5Gc antibodies that supposedly occur in humans [11, 14], and those had to be generated by immunization to allow their investigation in mice [11, 12]. Yet, in human studies, glycan microarray analysis revealed that certain anti-Neu5Gc antibodies can serve as a carcinoma biomarker [15] and that high levels of total anti-Neu5Gc IgG are associated with increased colorectal cancer risk, but not with red meat intake [16]. Altogether, the co-existence and interactions between Neu5Gc on cells with circulating anti-Neu5Gc antibodies have been suggested to modulate inflammatory response characteristics to meditate diseases [5, 17]; however, a direct correlation between anti-Neu5Gc antibodies and the diet in humans has been elusive.

Neu5Gc on human tissues and cells most likely originate from various dietary sources, given the absence of an alternative biosynthetic pathway to the CMP-Neu5Ac hydroxylase. Food items derived from mammals contain glycoproteins and glycolipids, many of which are covered with sialic acids. The two most common sialic acids in mammals are *N*-acetylneuraminic acid (Neu5Ac) and its hydroxylated form Neu5Gc, and their levels vary in different organisms and tissues [6]. While Neu5Ac is a native “self” carbohydrate in humans, Neu5Gc is a non-human immunogenic carbohydrate [17]. Neu5Gc is abundant in red meat and dairy, while scant in some fish, and non-existent in chicken [11, 18]. In this study, we investigated the dietary effects on the global burden of world colorectal cancer, and the effects of dietary Neu5Gc on the levels and repertoires of circulating anti-Neu5Gc antibodies in humans using the French NutriNet-Santé cohort based on detailed 24-h dietary records, in order to provide a mechanistic explanation for the cancer risk associated with red meat consumption. We further validated our findings by affinity purification of such antibodies and detailed glycan microarray analysis. Based on these findings, we developed tools to assess diet-related induction of anti-Neu5Gc IgG and tools for personalized dietary recommendations related to Neu5Gc intake.

## Methods

### Study participants and human serum samples

To investigate the relationship between dietary Neu5Gc and circulating anti-Neu5Gc antibodies in humans, we used the well-established NutriNet-Santé cohort (ClinicalTrials.gov # NCT03335644). This is a French web-based cohort study launched in 2009 with the objective to investigate the relationship between nutrition (nutrients, foods, dietary patterns, physical activity), health and diseases (e.g., cancer, cardiovascular diseases, metabolic syndrome, rheumatoid arthritis, and hypertension), and determinants of dietary behaviors and nutritional status [19]. At baseline and every 6 months, participants completed three non-consecutive validated web-based 24-h dietary records, randomly distributed between week and weekend days to take into account intra-individual variability, in which they declared all foods and beverages consumed during periods of 24 h. The mean dietary intakes from all the 24-h dietary records available during each participant’s follow-up were averaged and considered as usual dietary intakes in these analyses. The NutriNet-Santé web-based self-administered 24-h dietary records have been tested and validated against an interview by a trained dietitian [20] and against blood and urinary biomarkers [21, 22]. Participants used the dedicated web interface to declare all foods and beverages consumed during a 24-h period for each of the three main meals (breakfast, lunch, dinner) and any other eating occasion, with an accurate estimation of portion sizes [23]. Dietary underreporting was identified on the basis of the method proposed by Black, using the basal metabolic rate and Goldberg cutoff, and under-energy reporters (20.0% of the participants of the cohort) were excluded [24]. A subsample of 19,621 volunteer participants attended clinical consultations (69 sites throughout France), where blood samples were collected by trained technicians using a standardized protocol, to constitute the NutriNet-Santé Biobank. Of those, we selected individuals with at least six dietary records (16,149 individuals), for which quartiles of Neu5Gc daily intake were calculated. For serum sample detailed analysis, 120 individuals with at least 18 dietary records were selected and included 10 men and 10 women aged 45–60 per Neu5Gc intake quartile by gender (Q1–Q4; 80 samples), and 10 men and 10 women aged > 60 per quartile, from the first and fourth quartiles by gender (Q1 and Q4; 40 samples). Men and women were matched for age, education levels, and smoking habits.

### Human serum samples of patients with infectious mononucleosis (IMN)

Samples were collected as described [9]. Briefly, sera from 45 patients with infectious mononucleosis (IMN) were collected at the onset of the overt clinical symptoms of the disease from the University Hospital of Grenoble and Nantes between 2007 and 2014. The gender ratio was 25 females/20 males, and the average age was 24 years. Epstein-Barr virus (EBV) IMN was serologically confirmed by the detection of VCA IgM in the absence of anti-EBNA1 IgG. EBV serostatus was determined in the plasma with a DiaSorin LIAISON XL automat, using EBNA1 IgG, EBV-VCA IgG, and EBV VCA IgM kits (DiaSorin, Saluggia, Italy). Patients and samples were coded for anonymity. Samples from 43 normal individuals, matched for age (± 3 years) and gender (1/1 ratio), were obtained from the regional blood bank and from Nantes University Hospital in conformity with regulatory and ethical requirements. All patients and healthy donors signed an informed consent form for the use of the samples. Samples were used in accordance with the Helsinki Declaration and Tel Aviv University Institutional Review Board.

### Antibodies

The antibodies are horseradish peroxidase (HRP)-goat-anti-human IgG (Bio-Rad), purified human IgG, Cy3-goat-anti-human-IgG (H+L), and HRP-conjugated affinity-purified Fc-specific goat-anti-human IgG (Jackson ImmunoResearch).

### Homogenization of food samples

French food samples (Additional file 1: Table S1) were shipped frozen from France to Tel Aviv University and stored at − 80 °C. Samples were thawed, 50 mg of each food sample was weighed, incubated at − 80 °C for 2 h, then lyophilized for overnight. Dried samples were dissolved in 1 ml of lysis buffer (50 mM Tris-HCl pH 7.4, 5 mM MgCl_2_, 1 mM dithiothreitol, 1 mM phenylmethylsulfonyl fluoride), thoroughly vortexed for 30 s, put on ice, then sonicated with a probe sonicator (Sonic Dismembrator, Fisher Scientific) three times at a medium power, each for 10 s with 30-s intervals incubation on ice. Sonicated solutions were then inserted into a glass Dounce tissue grinder (2 ml; Sigma) and homogenized with a loose pestle then with a tight pestle (10 times each). The homogenate was centrifuged 10,000×*g* for 5 min to remove pelleted nuclei and cell debris, and protein content in the supernatant homogenate was evaluated by a standard BCA assay according to manufacturer’s protocol (Pierce). The homogenate was stored at − 20 °C until use.

### Sialic acid analysis by DMB-HPLC

Sialic acid (Sia) content in food homogenate samples was analyzed as described [25] with some modifications. Sias were released from glycoconjugates by acid hydrolysis with 0.1 M of H_2_SO_4_ for 1.5 h at 80 °C followed by neutralization with 0.1 M of NaOH [26]. Free Sias were derivatized with 1,2-diamino-4,5-methylenedioxybenzene (DMB; Sigma) for 2.5 h at 50 °C, separated by Microcon-10 centrifugal filters and analyzed by fluorescence detection on reverse-phase high-pressure liquid chromatography (DMB-HPLC) (Hitachi HPLC Chromaster). HPLC run was on C18 column (Phenomenex C18 Gemini 250 × 4.6 mm) at 24 °C in running buffer (84.5% ddH_2_O, 8.5% acetonitrile, 7% methanol) for 60 min at a flow rate of 0.9 ml/min. Quantification of Sias was done by comparison with known quantities of DMB-derivatized Neu5Ac [26].

### Quantification of Neu5Gc in food items

Neu5Gc content in French food items was measured by DMB-HPLC. All the food items containing animal products among the 3500 food items of the NutriNet-Santé food composition table were identified. Among them, Neu5Gc data were not available for 10 food items (roasted horse meat, horse steak, roasted deer, roasted doe, kid, roasted kid, roasted buffalo, Antwerp filet, mixed mince, and potjevleesch), originated from horse, buffalo, doe, and goat. These 10 food items had been consumed at least once by 6 participants among the 120 selected cohort. For these food items, a value resulting from the mean of the Neu5Gc content of beef and lamb was computed. Thus, food sources of Neu5Gc included meat (cow, lamb, goat, pig, rabbit, and bush meats) and dairy (cow, sheep, buffalo, and goat). Except for those missing items, Neu5Gc in all other food items in the questionnaires were directly quantified.

### Calculation of individual’s daily Neu5Gc intake

We used 19,621 participants enrolled between May 2009 and May 2015 in the NutriNet-Santé study, and the total amount of dietary Neu5Gc intake (μmol/day) was computed for each participant using all the available data on 24-h dietary records for each food source. Hence, for all available 24-h dietary records, food items containing Neu5Gc were identified (meat from cow, lamb, goat, pig, rabbit, horse, buffalo, doe, and goat; dairy from cow, sheep, buffalo, and goat), and a mean daily intake for each food item (in g/day) was computed for each participant. For this purpose, French recipes validated by food and nutrition professionals were used to assess the amounts of simple food items containing Neu5Gc (see the list above) consumed by the participants from composite dishes obtained through the 24-h dietary records. Then, daily Neu5Gc contribution of each food source (μmol/day) was calculated by multiplying the mean amount (g/day) consumed by the measured Neu5Gc concentration (μmol/g) in that food source.

### Measurements of anti-Neu5Gc IgG reactivity by enzyme-linked immunosorbent assays (ELISA)

Serum anti-Neu5Gc IgG reactivity was measure by three ELISA methods: [1] an ELISA inhibition assay (EIA assay) using coated wild-type (WT) mouse serum sialo-glycoproteins and *Cmah*^*−/−*^ as an adsorbent for non-specific reactivity [27]; [2] an ELISA using coated mouse serum glycopeptides (GP assay) [28]; [3] an ELISA inhibition assay using coated WT mouse serum sialo-glycopepetides (GP), with the same GP target as a competitive inhibitor, followed by deduction of the inhibited signal value with GP inhibitor from the native signal obtained without GP (GP-EIA assay) [28].

### ELISA inhibition assay (EIA)

Specific overall anti-Neu5Gc IgG reactivity in human sera was evaluated by an ELISA against coated mouse serum sialo-glycoproteins, as described [27]. Briefly, Costar 96-well were coated overnight at 4 °C with 1 μg/well WT pooled mouse sera (lacking mouse-anti-human IgG) in coating buffer (50 mM sodium carbonate-bicarbonate buffer, pH 9.5). Wells were blocked for 2 h at room temperature (RT) with PBS/OVA blocking buffer (PBS pH 7.3, 1% chicken ovalbumin). During the blocking, human serum was diluted 1:100 in EIA buffer (PBS pH 7.3, 1% chicken ovalbumin and *Cmah*^*−/−*^ pooled sera that lack mouse-anti-human reactivity, diluted at 1:4000) and incubated on ice for 2 h. Next, PBS/OVA was removed from the wells, and pre-incubated human serum was added to triplicate wells at 100 μl/well then incubated at RT for 2 h. Wells were washed three times with PBST (PBS pH 7.3, 0.1% Tween-20); detection antibody was then added (100 μl/well, 1:7000 HRP-goat-anti-human IgG diluted in PBS) and incubated for 1 h at RT. After washing three times with PBST, wells were developed with 0.5 mg/ml *O*-phenylenediamine in citrate-PO_4_ buffer, pH 5.5; reaction was stopped with H_2_SO_4_; and absorbance was measured at a 490-nm wavelength on a SpectraMax M3 (Molecular Devices).

### Glycopeptides ELISA (GP assay)

Anti-Neu5Gc IgG reactivity in human serum samples was evaluated against coated WT mouse serum glycopeptides by ELISA. Neu5Gc-positive glycopeptides (GP) were prepared from the serum of WT C57BL/6 mice, as described [28]. Costar 96-well were coated overnight at 4 °C with 150 pmol/well GP in coating buffer (50 mM sodium carbonate-bicarbonate buffer, pH 9.5). Wells were blocked for 2 h at RT with PBS/OVA. After removal of the buffer, 1:100 diluted human sera in PBS/OVA were added to triplicate wells at 100 μl/well then incubated at RT for 2 h. Wells were washed three times with PBST, detection antibody was then added (100 μl/well, 1:7000 HRP-goat-anti-human IgG diluted in PBS) and incubated for 1 h at RT. After washing three times with PBST, wells were developed with 0.5 mg/ml *O*-phenylenediamine in citrate-PO_4_ buffer, pH 5.5, reaction stopped with H_2_SO_4_, and absorbance was measured at a 490-nm wavelength on a SpectraMax M3 (Molecular Devices).

### Glycopeptides ELISA inhibition assay (GP-EIA assay)

Specific anti-Neu5Gc IgG reactivity in human serum samples was evaluated against coated WT mouse serum glycopeptides compared to competition with the same coated targets by ELISA (GP-EIA assay), as described [28]. Costar 96-well were coated overnight at 4 °C with GP at 150 pmol Sia/well in coating buffer (50 mM sodium carbonate-bicarbonate buffer, pH 9.5). Wells were blocked for 2 h at RT with PBS/OVA. During the blocking, human serum was diluted 1:100 in blocking buffer GP-EIA inhibition buffer (PBS pH 7.3, 1% chicken ovalbumin, GP at 0.03 mM Neu5Gc) and incubated on ice for 2 h. Next, PBS/OVA was removed from wells, and inhibited human serum was added to triplicate wells at 100 μl/well then incubated at RT for 2 h. Subsequently, wells were further processed as described (GP/EIA assay). To calculate specific anti-Neu5Gc IgG reactivity, the binding signal obtained with GP inhibitor was deducted from the signal obtained against coated GP without the inhibition [28].

### Affinity purification of anti-Neu5Gc antibodies from human sera

Antibodies were affinity-purified from pooled human serum samples (per Neu5Gc-consumption quartile, described in context) on sequential columns of human and chimpanzee serum glycoproteins, as previously described [10, 29]. Chimpanzee sera were obtained from the local zoo only during routine maintenance procedures and kindly provided by Dr. Gillad Goldstein, curator of the Zoological Center Tel Aviv, Safari Park (Israel), and Dr. Nili Avni-Magen, Head Veterinarian and Zoological Director of The Tisch Family Zoological Gardens in Jerusalem (Israel).

### Sialoglycan microarray analysis

Microarrays were fabricated with NanoPrint LM-60 Microarray Printer (Arrayit, CA) on epoxide-derivatized slides (Corning or PolyAn 2D) with 16 sub-array blocks on each slide (version 3). Slides were developed with the selected 120 human serum samples diluted 1:100 and analyzed as previously described [25, 30]. Briefly, slides were rehydrated with dH_2_O and incubated for 30 min in a staining dish with 50 °C pre-warmed 0.05 methanolamine in 0.1 M of Tris-HCl, pH 9.0 to block the remaining reactive epoxy groups on the slide surface, then washed with 50 °C pre-warmed dH_2_O. Slides were centrifuged at 200×*g* for 3 min, then fitted with ProPlate™ Multi-Array 16-well slide module (Invitrogen) to divide into the 16 sub-arrays (blocks). Slides were washed with PBST (PBS pH 7.4, 0.1% Tween-20), aspirated, and blocked with 200 μl/sub-array of PBS/OVA blocking buffer for 1 h at RT with gentle shaking. Next, the blocking solution was aspirated and 100 μl/ block of human serum diluted 1:100 in PBS/OVA was added, then slides were incubated at RT with gentle shaking for 2 h. Slides were washed three times with PBST then with PBS for 5 min/wash with shaking, then binding detected with 1.5 μg/ml Cy3-goat-anti-human IgG diluted in PBS at 200 μl/block, then incubated at RT for 1 h. Slides were washed three times with PBST, then with PBS for 5 min/wash followed by removal from ProPlate™ Multi-Array slide module which were immediately dipped in a staining dish with dH_2_O and were incubated for 10 min with shaking followed by centrifugation at 200×*g* for 5 min, then dry slides scanned immediately.

### Array slide processing

Processed slides were scanned and analyzed at 10 μm resolution with a Genepix 4000B microarray scanner (Molecular Devices) using 350 gain, as described [30]. Images were analyzed by Genepix Pro 6.0 software (Molecular Devices). Spots were defined as circular features with a variable radius, and local background subtraction was performed. Data were analyzed by Excel.

### Affinity equilibrium constant *K*_*D*_ calculation by microarray

The affinity of serum anti-Neu5Gc IgG against diverse Neu5Gc-glycans was analyzed by glycan microarray, as described [31]. Briefly, slides were developed as above at serial dilutions (a factor of 2) of affinity-purified pooled serum anti-Neu5Gc IgG antibodies ranging at 40–6.1 × 10^−4^ ng/μl (266.67–0.033 nM) in PBS/OVA blocking buffer. *K*_*D*_ is calculated by fitting a plot of response at equilibrium against a wide range of purified anti-Neu5Gc antibody concentrations (non-linear fit with one-site specific binding, GraphPad Prism 7.0).

### Statistical analyses

As nutritional habits and intakes vary across gender, we computed statistical analyses by gender. Apart from total Neu5Gc intakes, three additional classes of dietary Neu5Gc sources were computed: Neu5Gc from meat, Neu5Gc from dairy cow, and Neu5Gc from dairy sheep and goat. Sex-specific tertiles for dietary Neu5Gc intakes were computed for each class (total daily Neu5Gc intake and daily Neu5Gc intake from meat, from dairy cow, and from dairy sheep and goat combined).

Statistical analyses were performed by SAS 9.4®. Quantitative variables presented as means ± standard deviation (SD) or means ± standard error of the mean (sem) if normally distributed, or as medians (25th–75th percentiles) if not normally distributed, while qualitative variables were presented as percentages. Mann-Whitney *U*, median tests, or ANOVA allowed to compare the means and medians between the two groups, or more than two groups, of quantitative variables, while the chi-square test examined significant differences across two qualitative variables. We also studied the associations between variables derived from antibody measures (individual Neu5Gc- and Neu5Ac-glycans, linear combinations of Neu5Gc-glycans, and αGal), and tertiles of Neu5Gc intake (Neu5Gc sources in four variables: total, cow meat, dairy cow, and dairy sheep and goat) or Neu5Ac using the SAS® quantreg procedure (a non-parametric regression that compares the median values of the outcome variables across the tertiles of the predictive variables). We used median regression because Neu5Gc intakes were not normally distributed. All the tests were two-sided and the statistical significance set at 0.05.

## Results

### International colorectal cancer (CRC) and red meat intake correlate in different nations

**Fig. 1:**
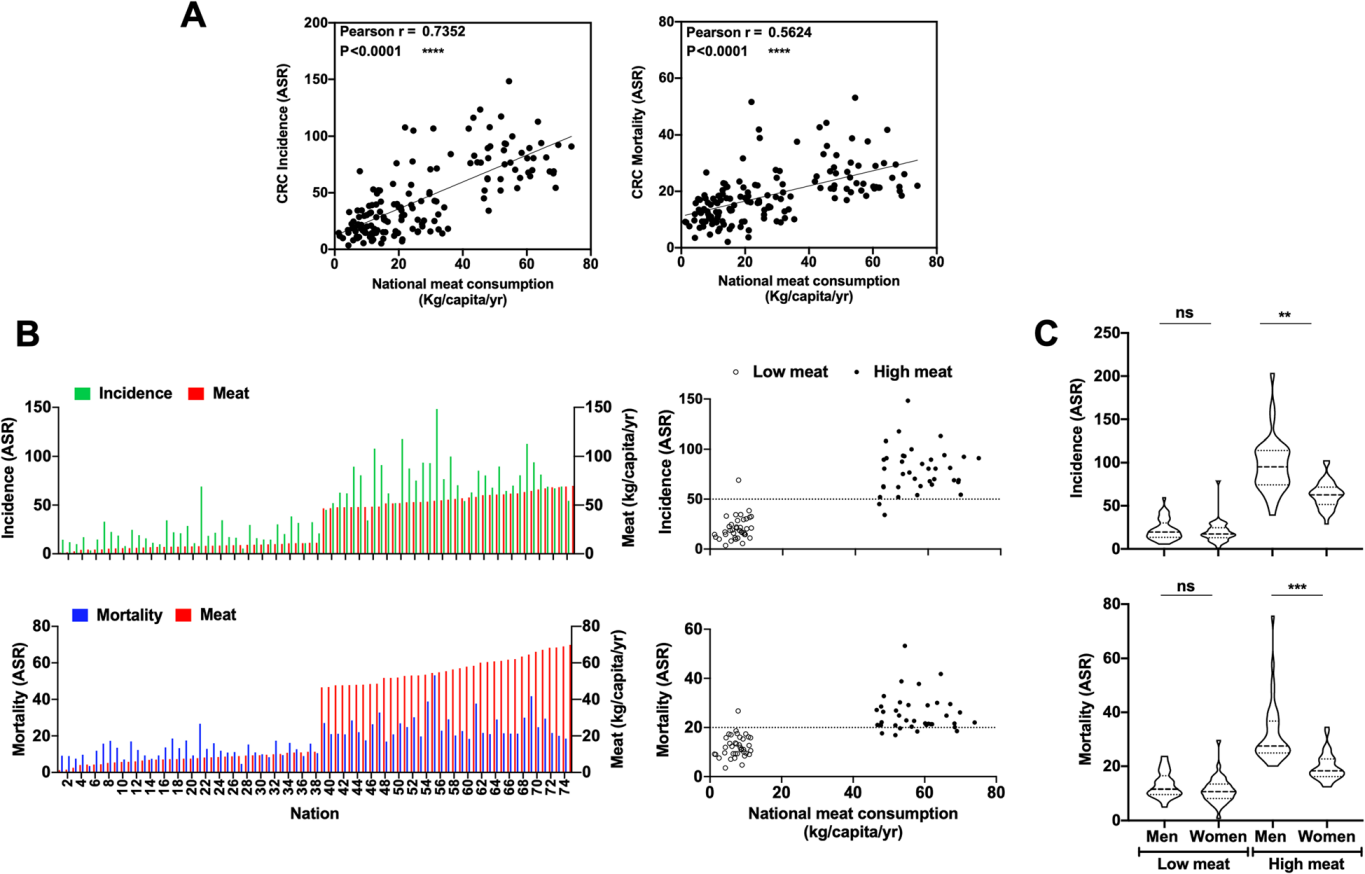
Global world data show the association between colorectal cancer (CRC) and red meat intake in different nations. **a** CRC incidence (Pearson *r* = 0.7352) and mortality (Pearson *r* = 0.5624) in different world nations (*n* = 152) strongly correlate with meat intake (both *p* < 0.0001). International CRC age-standardized incidence and mortality rates (ASR per 100,000 person-years, including colon, rectum, anus cancers) in individuals aged 45–69 from GLOBOCAN [34] and international per capita meat intake from FAOSTAT [33] (including bovine, mutton, goat and pig; excluding poultry and aquatic mammals; Additional file 2: Data file S1). **b** Distribution of CRC incidence (Pearson *r* = 0.8482) and mortality (Pearson *r* = 0.7249) per nation of the highest and lowest meat intake quartiles (*n* = 38 each) strongly correlates (both *p* < 0.0001). **c** CRC incidence and mortality per nation of the highest and lowest quartiles of meat consumption (*n* = 38 each) divided by gender show a strong correlation in nations with high levels of meat intake (Kruskal-Wallis test, **p < 0.0049 and ****p < 0.0001, respectively), but not in nations with low levels of meat intake

While many epidemiological studies support increased cancer risk with high meat intake, we wanted to explore this relationship at the national level, based on the available global national meat consumption and cancer risk data. Dietary habits can vary dramatically in different parts of the world [32]. To evaluate the effect of red meat intake on CRC in different world nations, national per capita meat intake from the Food and Agriculture Organization (FAO) of the United Nations, FAOSTAT database [33], and CRC age-standardized incidence and mortality rates from GLOBOCAN database [34] were extracted (Fig. 1; Additional file 2: Data file S1). Both CRC incidence and mortality positively correlate with meat intake (Fig. 1a). CRC rates were lowest in Sri Lanka, India, and many African nations, while highest in Australia, USA, Europe, and South American nations (Additional file 2: Data file S1), showing dramatic differences between the highest and lowest meat intake quartiles (Fig. 1b). Gender had no effect on CRC rates in nations of the lowest meat intake quartile (20.25 ± 1.17 g/capita/day, mean ± sem), but in nations of the highest meat intake quartile (156.4 ± 3.40 g/capita/day), there were higher incidence and mortality rates in men compared to women (Fig. 1c). These findings are consistent with international comparisons of cancer risk conducted on a limited number of nations > 40 years ago [35].

These striking age-standardized correlations do not account for other confounding factors (e.g., weight, physical activity, smoking, alcohol); however, there is vast literature supporting meat-related cancer risk, particularly in CRC, the third most common cancer worldwide [36–39]. As a result, meat was recently classified as carcinogenic by The International Agency for Research on Cancer [IARC; Consumption of processed meat was classified as carcinogenic (group 1), while consumption of red meat as probably carcinogenic to humans (group 2A)] [3]. Furthermore, in the third expert report on diet, nutrition, physical activity, and cancer of the World Cancer Research Fund (WCRF) and the American Institute for Cancer Research (AICR), strong evidence was found for the roles of processed meat and red meat in CRC risk, both judged to be “convincing” and “probable,” respectively [4]. The Continuous Update Project (CUP) from the leading authority WCRF is the world’s largest and most updated resource on cancer prevention, adjusted for body mass index (BMI or body fatness for some studies) and alcoholic drinks, thus excluding such confounding factors and strongly supporting the role of meat consumption in CRC risk [4]. The meat cancer risk had been partially explained by high-energy/fat Western diet, or various compounds in meat, such as N-nitroso compounds, salts, nitrates, nitrites, heme iron, saturated fat, estradiol, and trimethylamine–N-oxide (TMAO) produced by gut microbiome [5]. More recently, the non-human immunogenic carbohydrate Neu5Gc and the circulating antibodies against it in humans had also been suggested to contribute to meat-related cancer risk [3, 5], mostly relying on studies in mice.

France is among the top 15 nations of high meat intake (Additional file 2: Data file S1) with a confirmed meat-related risk of CRC [36] and breast cancer [40] even after adjustment of confounding factors such as alcohol consumption and BMI. In the French prospective NutriNet-Santé cohort study, red meat intake was associated with increased overall cancer risk (HR_Q5 vs. Q1_ = 1.31; 95% CI 1.10, 1.55; *p*_trend_ = 0.01) and increased breast cancer risk (HR_Q5 vs. Q1_ = 1.83; 95% CI 1.33, 2.51; *p*_trend_ = 0.002) [40]. We used the NutriNet-Santé cohort to further investigate the relationship between Neu5Gc and anti-Neu5Gc antibodies with meat and dairy intake in a qualitative and quantitative manner.

### Evaluating levels of daily Neu5Gc intake from red meat and dairy

**Fig. 2:**
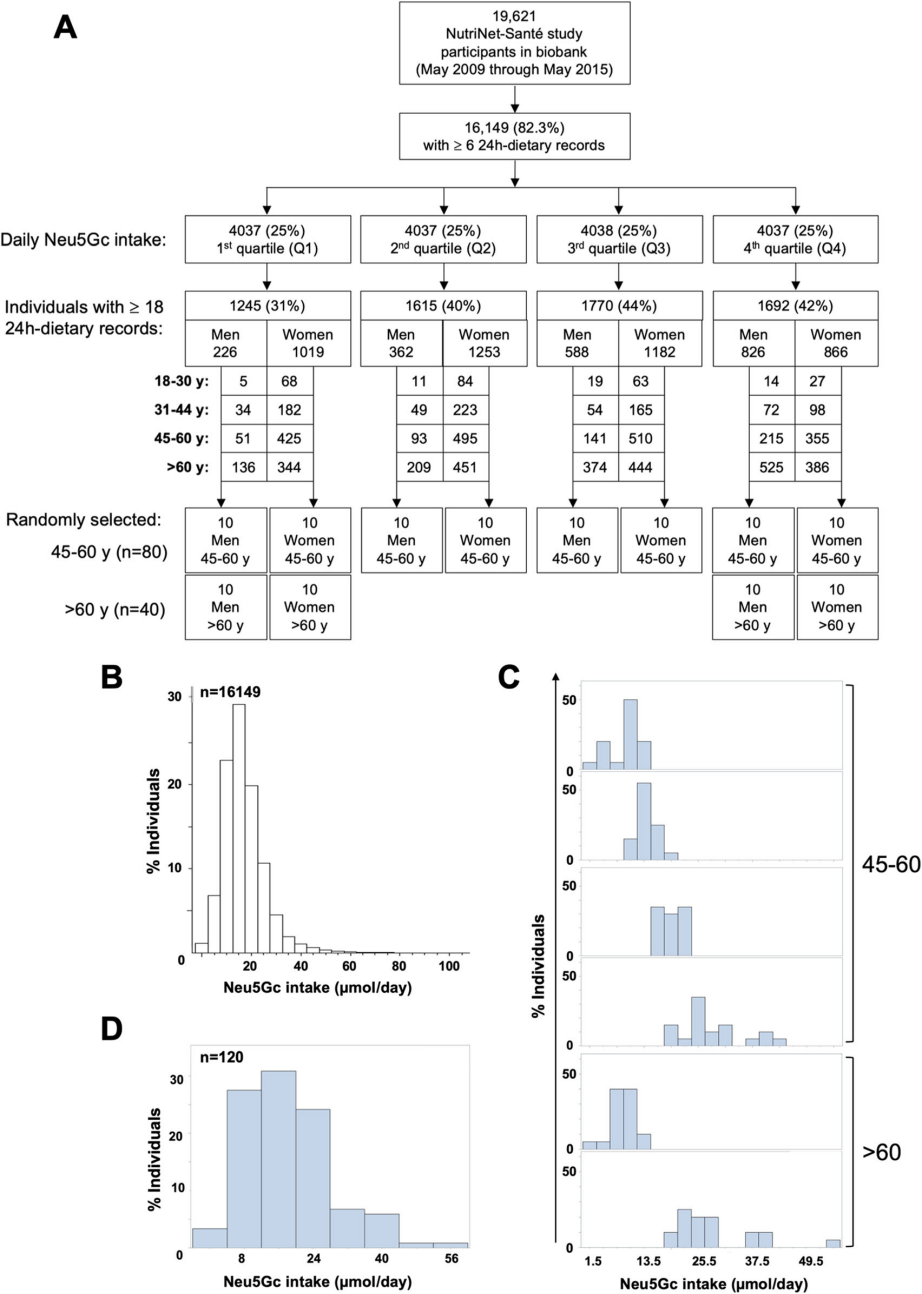
Daily Neu5Gc intake in the NutriNet-Santé study cohort. **a** Flow chart describing the selection of study cohort. **b** Distribution of the NutriNet-Santé study participants (May 2009 through May 2015) according to daily Neu5Gc intake calculated from the total mean Neu5Gc of 24-h dietary records for each individual. **c** Ten men and 10 women were selected per Neu5Gc intake quartile by gender (age 45–60, Q1–Q4; age > 60, Q1 and Q4), each with at least 18 dietary records. **d** Diversity of daily Neu5Gc intake in the selected 120 individuals (of 16,149 examined)

The amounts of Neu5Ac and Neu5Gc were accurately quantitated in diverse food items (Additional file 1: Table S1). On average, Neu5Ac content was ~ 3 times greater than Neu5Gc (414 ± 58 nmol/gr versus 149 ± 30 nmol/gr, respectively; mean ± sem). Neu5Gc content was highest in dairy sheep and goat products, moderate in red and processed meat, but rather low in dairy cow (422 ± 10 nmol/gr, 118 ± 17 nmol/gr, 21 ± 1 nmol/gr, respectively). Yet daily dietary Neu5Gc intake relies on actual amounts of food consumed by individuals (e.g., common beef steak serving size is ~ 225 g/day, while much lower for dairy). To account for individual records, daily Neu5Gc intake was calculated from all available NutriNet-Santé participants enrolled between May 2009 and May 2015 and that had a minimum of six 24-h dietary records (16,149 participants of 19,621 registered; Fig. 2a). Based on these questionnaires and Neu5Gc measurements in food, daily Neu5Gc intake was calculated per participant (Fig. 2b), then quartiles of total dietary Neu5Gc intakes were computed by gender and age (Q1–Q4; Additional file 1: Table S2; Fig. 2a).

**Fig. 3:**
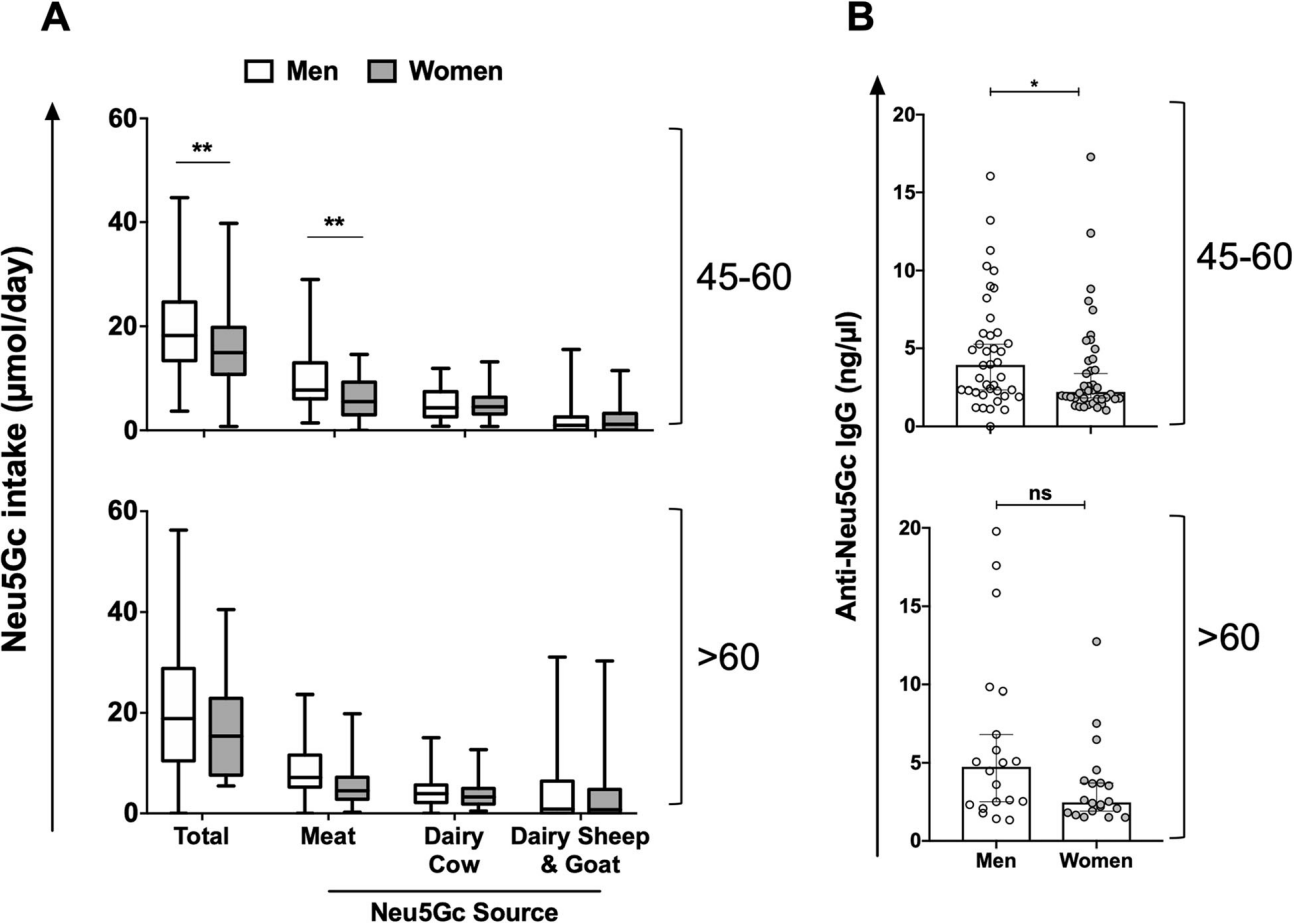
Distribution of daily Neu5Gc intake and anti-Neu5Gc IgG by age and gender. **a** Significantly higher total daily Neu5Gc intake in men compared to women (age 45–60; *n* = 40 per gender) mostly contributed from higher consumption of red meat. Similar trend in the group aged > 60 (*n* = 20 per gender; median and whiskers of min-max; two-way ANOVA with Bonferroni posttest; **p = 0.0015). **b** Overall anti-Neu5Gc IgG (by EIA) were significantly higher in men compared to women aged 45–60, with a similar trend in the group aged > 60 (median with 95% CI, Mann-Whitney test; *p = 0.0397; ns, *p* = 0.0822)

For further detailed analysis, 120 representative individuals who provided at least 18 dietary records, and had available blood samples, were randomly selected (Table 1; Fig. 2a). This focused cohort of 120 individuals included 10 men and 10 women aged 45–60 per Neu5Gc intake quartile by gender (Q1–Q4; 80 samples) and 10 men and 10 women aged > 60 per quartile, from the first and fourth quartiles by gender (Q1 and Q4; 40 samples) (Fig. 2a, c, d, Table 1). Selected men and women were matched for age, education levels, and smoking habits (Table 1), but nutritional habits and intakes were expected to vary across gender [41, 42]. Accordingly, in this cohort, there were statistically significant differences between men and women in energy intake and intake of proteins, animal proteins, lipids, and carbohydrates (Table 1). Hence, the total daily Neu5Gc intake in this study cohort was first computed by gender and age (Fig. 3a; Additional file 1: Table S2). Generally, daily Neu5Gc intake was largely contributed from cow’s dairy and meat (33% and 25%, respectively), then from pig’s meat (16%), goat’s dairy (13%), sheep’s dairy (11%), and lamb’s meat (2%). Hence, dietary Neu5Gc was also divided into three sub-classes based on the contributing food source (red meat, dairy cow, and dairy sheep or goat; Fig. 3a). Total daily Neu5Gc intake was significantly higher in men versus women aged 45–60, largely contributed due to higher consumption of red meat. Similar trends were found in the > 60 age group, though differences were not statistically significant (Fig. 3a). There were no significant differences in dairy consumption between men and women in both age groups.

**Table 1:** General characteristics of the study cohort of 120 representative individuals, each with at least eighteen 24-h dietary records (means ± SD for continuous variables; relative frequencies for qualitative variables)

Characteristics	Age group (years)					
	45–60			> 60		
	Men	Women	*p*	Men	Women	*p*
*N*	40	40		20	20	
Age, years	57.0 ± 4.5	58.0 ± 4.4	0.27	70.3 ± 4.6	69.2 ± 4.7	0.45
Educational level						
Primary or less	0	5	0.44	5	15	0.55
Secondary	37.5	42.5		30	30	
University	62.5	52.5		65	55	
Tobacco smoking						
Non-smokers	47.5	47.5	0.6	30	45	0.28
Ex-smokers	37.5	45		60	35	
Current smokers	15	7.5		10	20	
Energy intake, kcal/day	2411 ± 450	1774 ± 336	0.0001	2247 ± 387	1780 ± 252	0.0001
Proteins intake, g/day	94.1 ± 18.6	74.1 ± 16.9	0.0001	93.3 ± 18.4	74.0 ± 12.5	0.0004
Animal protein intake, g/day	62.1 ± 16.7	49.3 ± 16.0	0.0008	62.3 ± 18.1	49.5 ± 11.5	0.01
Lipids intakes, g/day	102.3 ± 23.0	74.9 ± 16.3	0.0001	92.6 ± 20.3	80.3 ± 18.1	0.051
Carbohydrates intakes, g/day	252.5 ± 64.8	189.6 ± 46.1	0.0001	226.2 ± 56.6	174.9 ± 33.9	0.001
Number of 24-h dietary records	21.4 ± 2.9	21.6 ± 3.3	0.83	21.7 ± 3.2	22.9 ± 3.4	0.26

### Gender differences in overall anti-Neu5Gc IgG response

It is not trivial to measure immune responses against Neu5Gc in light of the large diversity of Neu5Gc-neoantigens on diverse glycans, glycoproteins, and glycolipids, at different densities and combinatorial collections on cell surfaces [6, 28, 43]. To assess the humoral responses against Neu5Gc, serum samples of the selected cohort (Fig. 2c, Table 1) were initially analyzed for their specific overall anti-Neu5Gc IgG reactivities. This was done against a collection of multiple Neu5Gc-containing antigens, by three methods that differ in their target antigens and/or signal measurements (EIA, GP, GP-EIA). Hence, a total of 120 serum samples were quantitatively analyzed to detect overall anti-Neu5Gc IgG responses (Additional file 1: Figure S1A). Frequency of distribution of the overall anti-Neu5Gc IgG reactivity demonstrated that the sensitivity of EIA was much higher than that of GP or GP-EIA assays (2–4 times higher concentration range; mean 4.4 ng/μl in EIA, but only 2 ng/μl and 1.1 ng/μl in GP and GP-EIA, respectively; Additional file 1: Figure S1A). The direct comparison showed that while GP correlated with GP-EIA (both measured against Neu5Gc-glycopeptides), these two assays had no correlation with EIA (Additional file 1: Figure S1B). This was despite the fact that the same coated target glycans were used for serum antibodies binding in all three assays, only that the glycans were conjugated to carrier proteins (glycoproteins; EIA) or to carrier peptides (glycopeptides; GP/GP-EIA). Hence, in EIA assay, the glycans were presented in the context of the native Neu5Gc-glycoproteins, while in GP/GP-EIA, there was a much denser population of coated glycans targets. These differences likely affected the antibody binding in a way that only a fraction of the circulating antibodies was measured by GP/GP-EIA assays. These findings further support the reliability of the EIA assay for measurements of overall anti-Neu5Gc IgG responses. In addition, they highlight the importance of the presentation mode of glycans that can mediate the detection of different pools of antibodies within the sera, even against the same glycan targets.

Importantly, EIA overall anti-Neu5Gc IgG responses showed a clear gender difference, with almost twice as much higher levels in men compared to women (Fig. 3b). The medians in the 45–60 age group showed a 1.8-fold difference in men compared to women (men 3.94 μg/ml, 2.21 to 6.01; women 2.22 μg/ml in women, 1.77 to 4.28) and in the > 60 age group 1.9-fold difference (men 4.75 μg/ml, 2.37 to 8.88; women 2.47 μg/ml in women, 1.84 to 3.82). These higher anti-Neu5Gc IgG levels in men were associated with similar gender differences in daily Neu5Gc intake (Fig. 3a). Of note, the international correlation analysis showed increased incidence and mortality rates in men versus women in nations of high meat intake, but not in nations of low meat intake (Fig. 1c), supporting the hypothesis that higher meat intake leads to increased cancer risk due to higher Neu5Gc intake from meat that leads to higher levels of anti-Neu5Gc IgG.

### Glycan microarrays reveal specific anti-Neu5Gc IgG responses associated with diet

Neu5Gc is recognized as foreign in humans, although it differs from the corresponding human sialic acid Neu5Ac by only a single oxygen atom [6]. Consumed Neu5Gc incorporates into native human glycans, thereby replacing the “self” human sialic acid Neu5Ac and generating multiple and diverse “non-self” immunogenic Neu5Gc-glycans neoantigens [8, 15, 18]. To gain further insight into the full characteristics of the responses against Neu5Gc and the diet effects, serum samples of the selected cohort (Fig. 2c) were analyzed by glycan microarrays. These were fabricated with a diverse collection of Neu5Gc-glycan antigens and their matching pairs of Neu5Ac-glycans, each with a terminal primary amine that mediated their covalent conjugation onto epoxide-coated slides (Table S3).

**Fig. 4:**
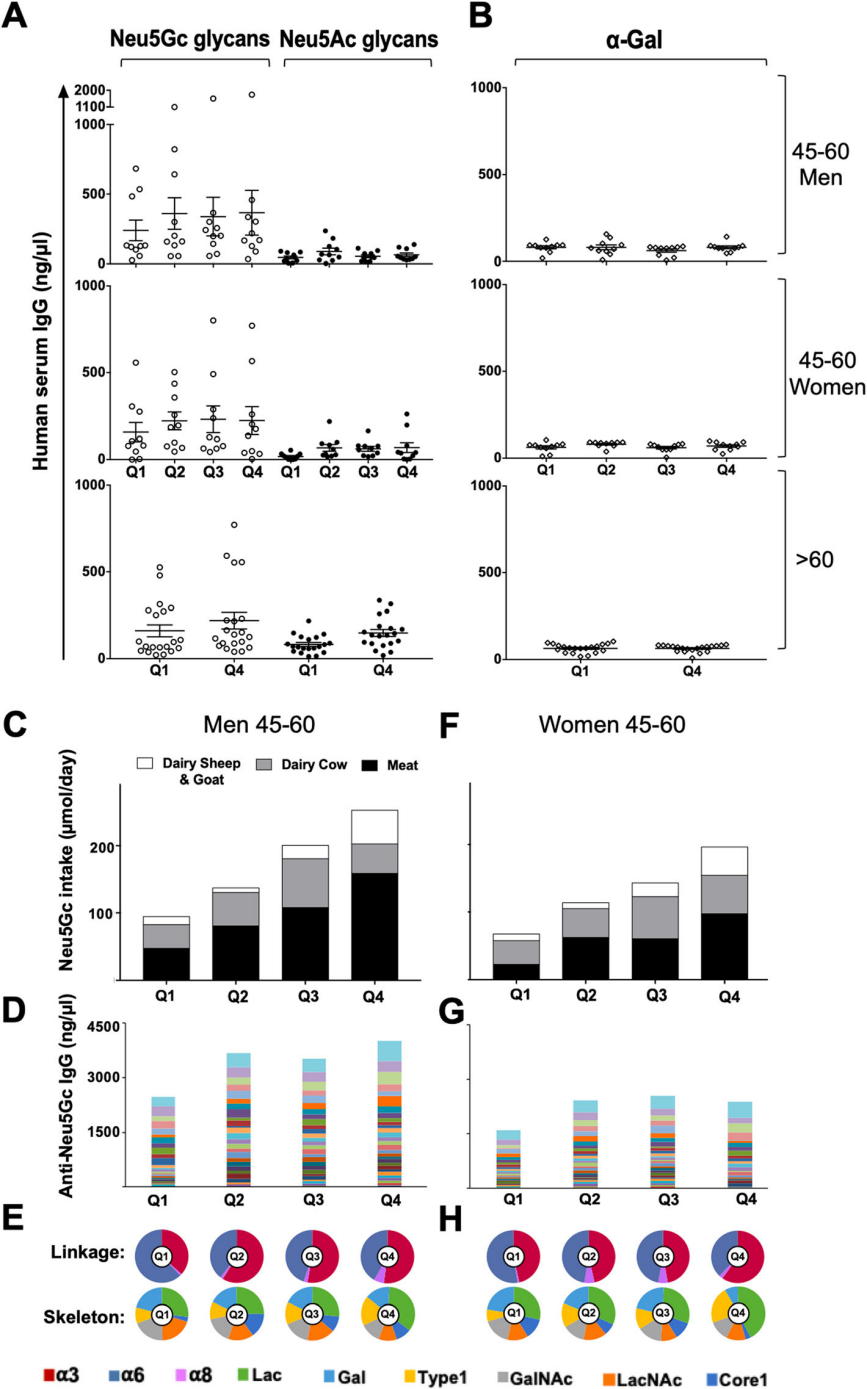
Glycan microarray analysis shows high anti-Neu5Gc IgG specificity and increased levels and diversity with higher Neu5Gc intake. Human serum IgG (*n* = 120; 1/100 dilution) detected with Cy3-anti-human IgG by glycan microarrays (24-pairs Neu5Gc-/Neu5Ac-glycans, αGal; Additional file 1: Table S3, Additional file 3: Data file S2). Relative fluorescence units (RFU) normalized to IgG (ng/μl) against printed standard curve/array [30]. **a** Sum serum IgG response/individual against Neu5Gc-/Neu5Ac-glycans, per Neu5Gc intake quartile, showed specific anti-Neu5Gc IgG (mean ± sem; Friedman ANOVA, *p* < 0.0001), a trend of elevated anti-Neu5Gc IgG at higher Neu5Gc intake. **b** Serum IgG/individual against αGal showed no change in levels/Neu5Gc intake quartile. **c** Men 45–60 stratified according to total Neu5Gc intake (Q1–Q4), contributing dietary sources plotted, showing increased Neu5Gc intake between quartiles, red meat dominant. **d** Men 45–60, antibodies/quartile show increased anti-Neu5Gc IgG levels between Q1 and Q2–Q4 (sum mean IgG response/glycan across individuals; different colors/specific Neu5Gc-glycan). **e** Pie charts of sum anti-Neu5Gc IgG response (**d**) divided per quartile according to reactivity against Neu5Gc-glycans with different Sia-linkages (Siaα2–3/6/8 linkages: α3, α6, α8, respectively) or underlying glycans [Lac (lactose; Galβ3Glc), Gal (galactose), type 1 (Galβ3GlcNAc), GalNAc (*N*-acetylgalactoseamine), LacNAc (*N*-acetyllactoseamine; Galβ4GlcNAc), core 1 (Galβ3GalNAcα)]. Differences in diversity at higher Neu5Gc intake, characterized by increased levels of α3-linked-Neu5Gc and Lac underlying glycans. **f** Women aged 45–60 stratified according to total Neu5Gc intake (Q1–Q4), increase between quartiles, similar contributions of Neu5Gc intake from red meat and dairy cow, dominance for dairy cow. **g** Women 45–60, anti-Neu5Gc IgG reactivity per quartile increase in levels between Q1 and Q2–Q4. **h** Women 45–60, pie charts of sum anti-Neu5Gc IgG response (**g**) divided by characteristic Neu5Gc-glycans linkage/skeleton, differences in diversity, as in men

Human serum IgG reactivity against each printed glycan was analyzed and quantified (Fig. 4; Additional file 3: Data file S2). Total IgG reactivity per donor was assessed as the sum of reactivities against all Neu5Gc-glycans versus all control non-immunogenic Neu5Ac-glycans, and samples divided according to quartiles of total daily Neu5Gc intake (Fig. 4a). In both men and women aged 45–60, the total reactivities against Neu5Gc-glycans appeared to be higher with elevated Neu5Gc consumption levels, while those against Neu5Ac-glycans were significantly lower and did not change by dietary quartile (Fig. 4a; *p* < 0.0001). Similar trends were observed in the group aged > 60, although Neu5Ac reactivities appeared to be higher than those of the group aged 45–60. Of note, the reactivity against Neu5Gc-glycans correlated with the reactivity against Neu5Ac-glycans only in the group aged > 60 of high Neu5Gc consumers (Q4; *R*^2^ = 0.74). The somewhat higher anti-Neu5Ac IgG in individuals > 60 likely represent cross-reactivity of anti-Neu5Gc IgG with Neu5Ac-glycans and fit the concept that the quality of immune responses deteriorates with age [44].

To further investigate the specificity of the Neu5Gc dietary effect, we evaluated the immune responses against another control immunogenic non-human sugar that shares common features with Neu5Gc. Similar to Neu5Gc, the αGal glycan antigen (Galα1-3Galβ1-4GlcNAc-R) cannot be synthesized by human cells due to a specific deletion in the gene encoding α1-3galactosyltransferase [45]. Yet, it is highly expressed by the gut microbiota [46], and as a result, all humans express circulating anti-Gal antibodies [45]. αGal is also expressed in mammalian derived food as red meat and dairy; however, once consumed, it is broken down into native human self-monosaccharides, as galactose. Thus, while both Neu5Gc and αGal are foreign antigens in humans, only Neu5Gc can incorporate into human cells through the diet and generate neoantigens [6, 47]. Given these differences, humoral responses against Neu5Gc-neoantogens are likely to be affected by the diet, in contrast to those against αGal. The αGal antigen was printed side-by-side with the Neu5Ac-/Neu5Gc-glycans on the microarrays and human serum IgG reactivity investigated per Neu5Gc dietary quartile (Fig. 4b). This analysis clearly showed that the anti-Gal reactivity did not change between quartiles in all groups, in contrast to the higher reactivity against Neu5Gc-glycans that was in fact related to elevated consumption of Neu5Gc. Thus, while dietary Neu5Gc had no effect on serum IgG responses against native Neu5Ac-glycans nor antigenic αGal glycan, serum anti-Neu5Gc IgG are highly specific and positively affected by dietary Neu5Gc.

### Higher anti-Neu5Gc IgG levels and altered diversity associated with Neu5Gc intake

For a detailed assessment of the dietary effects on antibodies characteristics, anti-Neu5Gc IgG were evaluated in individuals aged 45–60 per quartile of total Neu5Gc intake by gender (Fig. 4). In both men and women, the total Neu5Gc intake was gradually elevated between quartiles, with much higher levels in Q4 (Fig. 4c, f). Men consumed almost twice as much meat than dairy, most obvious in Q4 (Fig. 4c and Additional file 1: Figure S2; consumed food items detailed in Additional file 1: Table S1). In fact, there was a significant overlap between men divided into quartiles based on total Neu5Gc consumption or based on Neu5Gc from meat (overlap was 63% ± 10%). Women appeared to consume more dairy than meat (Additional file 1: Figure S2), reaching comparable levels in Q4 (Fig. 4f). Hence, Neu5Gc was dominantly contributed from red meat in men, while dairy in women.

To gain further insight into the effects of higher Neu5Gc intake on antibodies, sera were examined by sialoglycan microarrays against 24 different Neu5Gc-glycans, and the sum of anti-Neu5Gc IgG levels assessed per quartile (Fig. 4d, g). This detailed analysis revealed that anti-Neu5Gc IgG responses were higher in men compared to women. These results corroborate the estimated overall anti-Neu5Gc IgG responses obtained with the EIA assay against diverse natural Neu5Gc-glycoproteins, which also showed significant differences with higher antibody levels in men compared to women (Fig. 3b). This correlation between EIA measurements and the sum of the detailed antibody reactivities measured against individual glycans by microarray suggests that the EIA can provide a reliable assessment of the levels of anti-Neu5Gc IgG at large. In both men and women, the sum of anti-Neu5Gc IgG was higher in Q2 compared to Q1 and remained high through Q4 (Fig. 4d, g). The differences in antibody levels were more prominent in men, likely owing to their much higher Neu5Gc intake compared to women (Fig. 3a). In fact, when men were stratified according to quartiles of Neu5Gc consumption from red meat, their dominantly contributing food source, anti-Neu5Gc IgG levels seemed to be almost twice as much in Q2–Q4 compared to Q1 (Additional file 1: Figure S3A). Similarly, dividing women according to quartiles of Neu5Gc intake from dairy cow showed higher antibodies levels in Q3–Q4 compared to Q1–Q2 (Additional file 1: Figure S3C). Therefore, despite the difficulties in assessing the direct contribution of each food source on antibody levels, the intrinsic differences between the dietary habits of French men versus women allowed to highlight the observation that higher total Neu5Gc, either from red meat or from dairy, contributes to higher levels of circulating serum anti-Neu5Gc IgG, thus further supporting the contribution of Neu5Gc/anti-Neu5Gc IgG to the observed international meat cancer risk correlations.

**Fig. 5:**
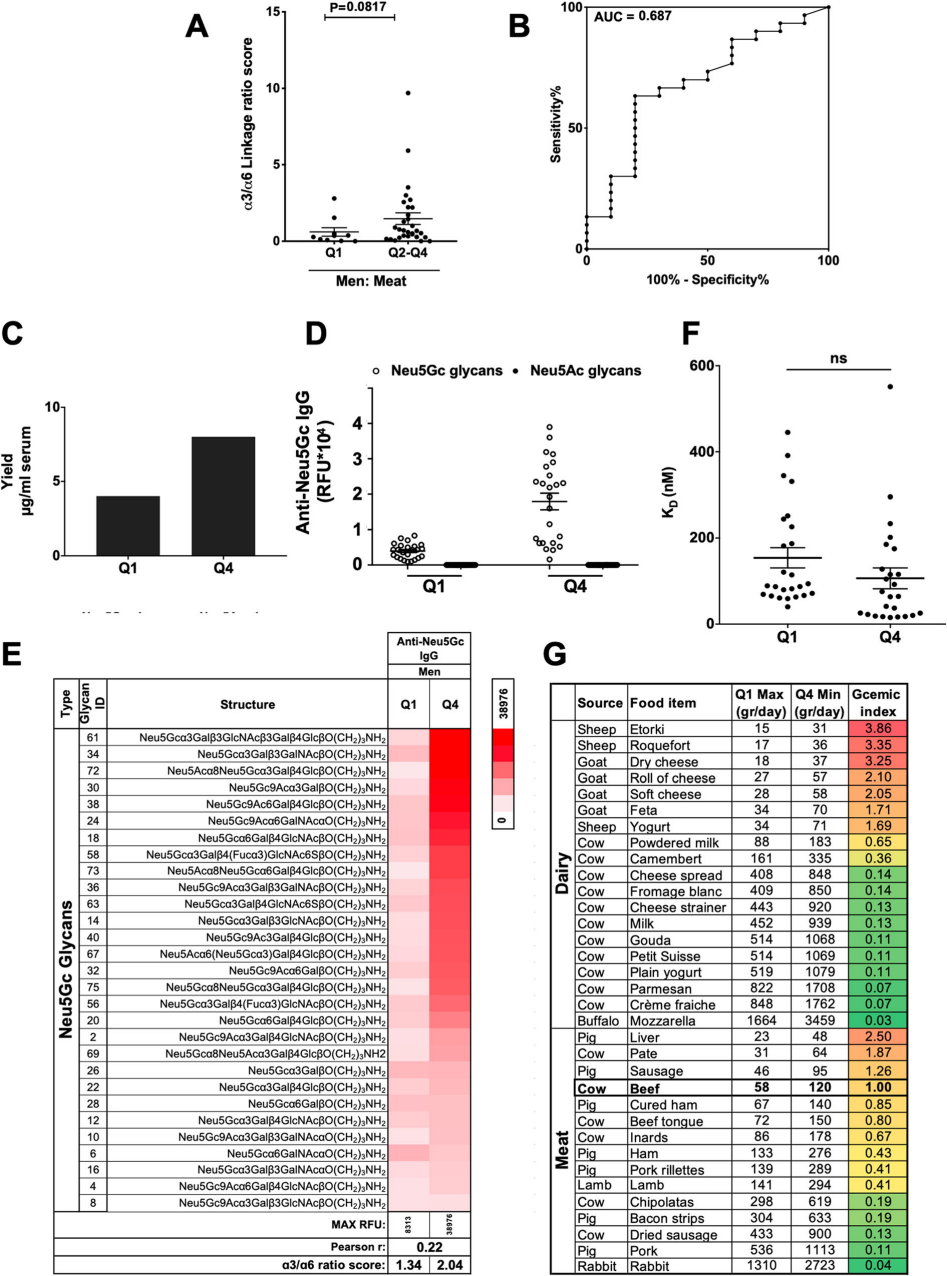
Characteristics of anti-Neu5Gc IgG and Neu5Gc in food. **a** Ratio between sum anti-Neu5Gc IgG against α3-linked-/α6-linked-Neu5Gc-glycans calculated in men 45–60 stratified based on meat Neu5Gc intake (Fig. S3). The α3/α6 linkage ratio in Q1 (*n* = 10) is lower than that in Q2–Q4 (*n* = 30) (Mann-Whitney test; *p* = 0.08; Q1 0.6080 ± 0.2806, Q2–Q4 1.472 ± 0.3722; mean ± sem). Similar comparing Q1 and Q4 (*p* = 0.09; Q1 0.6080 ± 0.2806, Q4 1.384 ± 0.3961). **b** ROC curve of α3/α6 linkage ratio score in Q1 (*n* = 10) compared to Q2–Q4 (*n* = 30) showed AUC 0.687 ± 0.098 (*p* = 0.08). Similar between Q1 and Q4 (*p* = 0.08, AUC 0.73 ± 0.1162). **c** Anti-Neu5Gc antibodies were affinity-purified from pooled sera of men 45–60 consuming low/high Neu5Gc from meat (Q1 5.39 ml and Q4 6.8 ml sera; *n* = 10 per group). Antibody yield was higher in Q4 than Q1 (8.01 versus 4.01 μg/ml serum, respectively). **d**, **e** IgG reactivity on sialoglycan microarrays (2 μg/block; detected with Cy3-anti-human IgG) revealed high specificity against Neu5Gc-glycans, no reactivity against Neu5Ac-glycans (**d**; each dot is IgG response/glycan), with altered diversity of glycans in Q4 over Q1 (**e**; Pearson *r* = 0.22). The α3/α6 linkage ratios: Q1 1.34, Q4 2.04. **f** Affinity (*K*_*D*_) per glycan calculated from anti-Neu5Gc IgG on microarrays at 16 serial dilutions (40–4.9 × 10^−3^ ng/μl; 266.7–0.033 nM; non-linear fit with one-site specific binding), showing no change in affinities with higher Neu5Gc intake (mean ± sem; *t* test). **g** Gram of food to consume to reach daily nmol Neu5Gc per quartile based on Neu5Gc content (measured by DMB-HPLC). Q1 max value based on gr food to reach men Q1 average of 9443 nmol/day. Q4 min value is based on gr food to reach women Q4 average of 19,627 nmol/day. *Gcemic index* is the Neu5Gc content (nmol/gr) in each food item relative to the amount measured in beef (163 nmol/gr)

To assess the impact of Neu5Gc intake on the repertoire of anti-Neu5Gc IgG, serum IgG reactivities against the different 24 Neu5Gc-glycans were stratified according to common features, such as linkages to underlying glycans (Siaα2–3/6/8 linkage; α3, α6, α8, respectively) and underlying glycan skeletons (Lac, Gal, Type 1, GalNAc, LacNAc, Core 1; Fig. 4; Additional file 1: Table S3). In both men and women, lower Neu5Gc intake showed higher levels of anti-Neu5Gc IgG reactive against Neu5Gcα2–6-linked glycans (α6), while higher Neu5Gc intake rather showed higher reactivities against Neu5Gcα2–3-linked glycans (α3; Fig. 4e, h). Similar changes were noticed when quartiles were divided according to the dominant food source in men or women (Additional file 1: Figure S3). In fact, linkage recognition had gradually shifted from α6 to α3 as Neu5Gc intake increased (Fig. 4, Additional file 1: Figure S3). Similarly, there was a gradually increased shift in recognition of Neu5Gc-glycans with underlying lactose (Lac) skeleton and to a lesser extent with underlying type 1 (Fig. 4, Additional file 1: Figure S3). Interestingly, in men 45–60, calculating the ratio score of anti-Neu5Gc IgG reactivity against Neu5Gc-glycans with α3-linkages over α6-linkages seemed to differentiate between those who consumed low red meat with those of higher Neu5Gc intake from red meat (Fig. 5a, b). Altogether, these changes suggest that irrespectively of gender or dominant Neu5Gc food source, higher Neu5Gc intake was associated with altered diversity of anti-Neu5Gc IgG, specifically shifting towards recognition of Neu5Gcα2–3-linked glycans, mostly associated with increased underlying lactose skeleton. Of note, both lactose and α3-linkage are features most commonly found with sialic acid-containing glycolipids (gangliosides), although they can also be found on glycoproteins [6].

To evaluate the specificity of the Neu5Gc diet effects on anti-Neu5Gc antibody levels and diversity, we used control samples to investigate whether these differences were also observed in sera from individuals with known elevated levels of anti-Neu5Gc IgG, but were not related to diet. It had previously been shown that during acute clinically overt Epstein-Bar virus (EBV) primo-infection (infectious mononucleosis (IMN)), the levels of anti-Neu5Gc IgG are significantly increased compared to healthy controls matched for age and gender, as measured by EIA assay [9]. To further evaluate changes in anti-Neu5Gc IgG repertoire in this situation, we obtained samples from the same cohort of 45 IMN patients and from 43 age/gender-matched healthy individuals (Additional file 1: Figure S4A) and examined those on sialoglycan microarrays (Additional file 1: Figure S4B-D). This analysis confirmed the increase in anti-Neu5Gc IgG levels also by microarray (Additional file 1: Figure S4B) demonstrating high specificity against Neu5Gc-glycans with no recognition of Neu5Ac-glycans (Additional file 1: Figure S4C). There were no differences in the repertoire between IMN patients and controls (Additional file 1: Figure S4D), despite the increased levels of antibodies and their high specificity. In fact, the ratios between reactivities against Neu5Gc-glycans with the different linkages (α3, α6, α8) had not changed at all. These findings are in clear contrast to the changes in anti-Neu5Gc IgG linkage diversity observed with high Neu5Gc diets, in which there is higher recognition of α3-linkage as Neu5Gc intake increase, replacing the preferred α6-linkage at lower Neu5Gc intake (Fig. 4, Additional file 1: Figure S3). These data provide strong evidence that Neu5Gc diet can specifically affect the levels and repertoire diversity of circulating serum anti-Neu5Gc antibodies, in both men and women, irrespectively of the dietary source contributing to higher Neu5Gc intake.

### Higher Neu5Gc diet is not associated with increased affinities of anti-Neu5Gc IgG

We have previously shown that human immunization with a biological drug containing Neu5Gc-glycoproteins (i.e., anti-thymocyte globulin (ATG), commonly used as an immunosuppressive treatment) can cause a vigorous and transient increase in anti-Neu5Gc IgG responses, with an altered repertoire [48]. Such drug-elicited increased anti-Neu5Gc IgG levels were also associated with higher affinities of the developed antibodies at peak of response, some also acquiring new specificities [48]. To examine whether Neu5Gc diet-induced anti-Neu5Gc IgG also acquire higher affinities, as in the case of drug-induced antibodies, serum anti-Neu5Gc antibodies from Q1 and Q4 by gender and age were affinity-purified. Due to limited volumes of samples, sera were pooled. Men aged 45–60 were divided according to Neu5Gc intake from red meat, then 10 serum samples per quartile were pooled and anti-Neu5Gc antibodies affinity-purified on sequential columns of Neu5Ac-glycoproteins and Neu5Gc-glycoproteins, then eluted from the latter with free Neu5Gc. The yield of affinity purification clearly demonstrated twofold greater antibodies levels in Q4 compared to Q1 (Fig. 5c), which is in complete agreement with the observed higher levels and reactivities found by serum analysis (Fig. 4). Moreover, the purified antibodies were highly specific against Neu5Gc-glycans with no recognition of any of the Neu5Ac-glycans (Fig. 5d), suggesting that the minor reactivity observed by serum analysis (Fig. 4a) is likely related to cross-reactive antibodies. Detailed glycan microarray repertoire analysis also demonstrated a clear change in the diversity of anti-Neu5Gc IgG, with preference towards reactivity against Neu5Gc-glycans with α3-linkage in Q4 (Fig. 5e).

To test whether the increased levels and reactivities of anti-Neu5Gc IgG were also associated with enhanced binding strength, the affinity equilibrium constant *K*_*D*_ was measured. Due to limited sample obtained by affinity purification, *K*_*D*_ was measured by glycan microarray (rather than kinetic measurements), against a constant concentration of each of the printed Neu5Gc-glycans (at 100 μM), by fitting a plot of response at equilibrium against a wide range of concentrations of the purified antibodies. This analysis revealed no significant differences in the affinities of the antibodies purified from Q4 compared to Q1, despite the much higher yields, although a slight and non-significant decrease in *K*_*D*_ was noticed (Fig. 5f). Similar results were obtained from affinity-purified antibodies of women aged 45–60 with quartiles divided according to dairy cow, revealing ~ 1.3-fold higher antibody yield in Q4 compared to Q1 (Additional file 1: Figure S5A). Analysis of the affinity-purified antibodies on glycan microarray demonstrated high specificity against Neu5Gc-glycans (Additional file 1: Figure S5B), with altered anti-Neu5Gc IgG repertoire (Additional file 1: Figure S5C), but no change in affinities of these antibodies (Additional file 1: Figure S5D). Interestingly, in these affinity-purified antibodies profiles, the ratio scores of anti-Neu5Gc IgG reactivity against Neu5Gc-glycans with α3-linkages over α6-linkages were 1.34 in Q1 and 2.04 in Q4 in men aged 45–60 (Fig. 5) and 1.98 in Q1 and 3.03 in Q4 in women aged 45–60 (Additional file 1: Figure S5). Altogether, these data provide, for the first time, direct evidence that dietary intake of Neu5Gc from meat and dairy have significant immunological consequences affecting anti-Neu5Gc antibodies in humans. Both serum samples and affinity-purified antibodies showed much higher levels of anti-Neu5Gc IgG antibodies with greater Neu5Gc intake, irrespectively of the contributing food source, as well as a change in diversity, but no change in affinity. Meat seems to be the dominant contributing dietary factor, at least in men, and is generally consumed at a larger serving size compared to dairy.

To translate these findings into practical personalized dietary recommendations, we calculated the gram of each food item that needs to be consumed to reach the ranges of average Neu5Gc consumed in the highest and lowest quartiles for both genders (Additional file 1: Table S4). The Q1 range is 6937–9443 nmol/day (min–max is Q1-women–Q1-men), and Q4 range is 19,627–25,265 nmol/day (min–max is Q4-women–Q4-men). In addition, we developed a *Gcemic index* as an easy tool to estimate the relative Neu5Gc content in different food items, based on the Neu5Gc content (nmol/gr) in each food item relative to the amount measured in beef (163 nmol/gr). A Gcemic index of 1 means that daily consumption of 58 g of beef at most is the maximal Q1-Neu5Gc (9443 nmol/day), while daily consumption of at least 120 g is the minimum Q4-Neu5Gc (19,627 nmol/day; Additional file 1: Table S4; Fig. 5g). Thus, one can consume 1–2 medium-sized beef steaks (225 g raw meat) per week to fall in Q1 range, while 4–5 steaks weekly is already at Q4 range. A lower Gcemic index (or high inverse Gcemic index; Additional file 1: Table S4) means higher grams of food consumed to reach the Q1/Q4 ranges. For example, the Mozzarella Gcemic index is 0.03 hence almost 30 times more grams can be consumed compared to beef, while the Roquefort Gcemic index is 3.86 suggesting consuming only ¼ the amount of beef can reach Q1/Q4 range (Additional file 1: Table S4; Fig. 5g). In general, cow dairy has the lowest Gcemic index, while sheep/goat dairy has the highest, while variable in meat. Arbitrarily dividing international meat cancer risk according to national intake of above/below 120 g meat daily (lowest beef amount in Q4) shows an increase of 3-fold in incidence and 2.5-fold in mortality in nations that consume > 120 g meat per day (Additional file 1: Figure S6). Interestingly, countries of high beef meat intake fall among the top 15 CRC incidence and mortality rates, including the USA, Australia, and France, as well as many counties in South America such as Argentina, Brazil, Uruguay, and Chile. While the Gcemic index can provide a simple estimate on Neu5Gc content in food, a direct correlation between specific amounts of consumed food with cancer risk requires further investigation, to account for other common risk factors.

## Discussion

To the best of our knowledge, this study provides the first experimental evidence of an association between an immunogenic carbohydrate dietary component and induction of serum antibodies against it, other than in allergy. In fact, there is a distinct dose-dependent positive association between Neu5Gc and circulating anti-Neu5Gc antibodies. High levels of anti-Neu5Gc IgG have been suggested to increase colorectal cancer risk in humans [16]. Thus, the international positive correlation between dietary meat and higher CRC incidence and mortality could possibly be mediated by an increase in anti-Neu5Gc antibodies and should be further established. Glycan microarray analysis provides a characteristic score (α3/α6-linkages response ratio) associated with high dietary Neu5Gc intake, and the developed *Gcemic index* provide a straightforward tool to assess the amount of Neu5Gc in diet and could be developed into personalized recommendations for specific patients at risk or for a general healthy lifestyle.

One of the limitations of this study is that the detailed analysis was based on the French population and dietary habits; however, it fits with international trends. In addition, the international per capita meat intake from FAOSTAT [33] is based on national food accounts (food balance sheets); therefore, these data cannot be used to determine the distribution of food that is available for consumption spatially, seasonally, or by demographic characteristics. Given the potential clinical applications of the diet-antibody on disease risk, it would be interesting to evaluate such associations in other specific populations with detailed meat consumption records.

Of note, in response to the latest evidence on meat and cancer risk [4], the World Cancer Research Fund International (WCRF) also clarified that “red meat can contribute to a healthy, balanced diet, as it is a good source of nutrients such as protein, iron, zinc and vitamin B12. Processed meat on the other hand has less valuable nutrients and can be high in fat and salt”. Current knowledge on meat cancer risk had been partially explained by Western diet rich in energy and fat, or by various compounds in meat [5], including heme [49]. While this iron-containing porphyrin functions in vital biological processes (i.e., oxygen transport, energy production, drug metabolism), heme can be toxic at high levels. Tumor cells exploit heme to modulate their energetic metabolism, to interact with the microenvironment, and to sustain proliferation and survival [49]. In addition, modern cooking methods had been suggested to generate mutagens like heterocyclic amines (HCAs) and polycyclic aromatic hydrocarbons (PAHs) in meat that could mediate its carcinogenic properties [50]. The type of meat, temperature, and duration of cooking directly affect the formation of such mutagens [51, 52]. However, none of the suggested mechanisms by which these compounds affect cancer (i.e., oxidative stress, inflammation, cytotoxicity, and perturbations to the normal process of apoptosis) is supported by sufficient evidence to confirm a mechanistic link between red meat intake and CRC risk [53]. Here, we propose a new element contributing to cancer risk in the form of the dietary carbohydrate antigen Neu5Gc and the antibodies humans generate against it, which particularly correlate with the dose of consumed Neu5Gc.

This research has several strengths. Importantly, the effect of diet on antibody responses was specific to Neu5Gc and could not be detected in response to other control dietary carbohydrates, Neu5Ac, and αGal. Neu5Ac is expressed on human cells but is considered to be a tolerized self-moiety [17], while αGal is only expressed by bacteria in the gut [46, 54], and even if consumed could be cleaved into the non-immunogenic galactose. The novelty in the developed methodology is several fold: (a) the use of a defined cohort with multiple, detailed and well-dispersed online 24-h dietary records, in both men and women, from diverse age groups; (b) accurate assessment of consumed Neu5Gc quantities from common food items; and (c) several improved quantitative methods to measure and characterize anti-Neu5Gc antibodies that include a standard curve in both EIA and glycan microarrays, which provides internal quality control and facilitates accurate measurements independent of experimental day and conditions. Furthermore, affinity purification of serum antibodies further corroborated the findings.

Neu5Gc in the diet affect the levels and diversity of serum anti-Neu5Gc antibodies, in both men and women, irrespectively of the dietary source that contributes to the higher Neu5Gc intake. There is extensive evidence in mice regarding the role of anti-Neu5Gc antibodies (elicited by immunization) in aggravating various chronic inflammation-mediated diseases [5, 7, 11, 13, 17, 55]. It is worth emphasizing that current evidence does not support Neu5Gc as a causative agent but rather one that contributes to the promotion and worsening of such diseases. The xenosialitis mechanism in human-like Neu5Gc-deficient mice had been shown to increase the incidence of hepatocellular carcinomas [11] and promote cancer growth [12], in an anti-Neu5Gc antibody dose-dependent manner [56]. It had also been suggested to exacerbate vascular endothelium inflammation [13]. On the other hand, there is limited information regarding such a disease mechanism in human patients. Our findings provide a new perspective and tools for the study of diet-related disease risk in humans, especially in cancer and cardiovascular disease.

Studies have shown that treatment with Neu5Gc-containing drug (rabbit anti-thymocyte globulin (ATG)) cause unintentional Neu5Gc immunization, leading to drug-elicited anti-Neu5Gc IgG of higher levels and altered repertoire [48, 57]. Most importantly, drug-induced anti-Neu5Gc IgG have increased affinity against diverse Neu5Gc-glycans [48], and ATG induction treatment in kidney transplant recipients was not associated with increased colon cancer risk [58]. In addition, drug-induced anti-Neu5Gc antibodies seemed to be different than the diet-induced antibodies, showing differential effects on Neu5Gc-expressing primary human endothelial cells that do not support inflammation circuits in vitro [59]. However, studies in human-like Neu5Gc-deficient mouse models showed a cancer therapeutic potential of anti-Neu5Gc antibodies when induced by immunization and treated with passive transfer [15, 56] or active vaccination [60], as a function of their dose and quality/affinity, supported by evidence of inverse hormesis effects of an optimal immune response curve [56]. Altogether, these accumulating evidences suggest that not all human anti-Neu5Gc antibodies are alike and the outcome of their effects on the disease can diverge from disease promotion to rather disease reduction and therapy. Apparently, these opposing effects could be related to both their induction source (elicited by diet or by immunization) and to the eventual quality of immune response.

## Conclusions

The described experiments provide ample evidence that Neu5Gc consumption from red meat and dairy can dictate the eventual levels, repertoire, and characteristics of circulating anti-Neu5Gc antibodies in humans. Given the association between red meat and cancer, these studies warrant further investigation into the role of Neu5Gc and anti-Neu5Gc antibodies into the risk of human diseases, including cancer.

## Supplementary information

**Additional file 1: Figure S1.** Measurements of anti-Neu5Gc IgG in 120 study cohort by ELISA. **Figure S2.** Distribution of Neu5Gc intake by food source. **Figure S3.** Increased levels and diversity of anti-Neu5Gc IgG with higher Neu5Gc intake. **Figure S4.** Anti-Neu5Gc IgG response in patients with infectious mononucleosis and controls. **Figure S5.** Characteristics of affinity-purified anti-Neu5Gc antibodies of women 45-60. **Figure S6.** International cancer risk according to national meat intake. **Table S1.** Sialic acid content (Neu5Ac and Neu5Gc) in common French food items measured by DMB-HPLC. **Table S2.** Daily Neu5Gc intake in NutriNet-Santé participants (May 2009 through May 2015) with a minimum of six 24-hour dietary records (total 16,149 participants). **Table S3.** List of glycans printed on glycan microarray and their characteristics. **Table S4.** Gcemic index.

**Additional file 2: Supplementary data file S1.** National world meat and cancer.

**Additional file 3: Supplementary data file S2**. Glycan microarray.

## Data Availability

Data are available in the supplementary files.
