# Supplementary material for: Association between Neu5Gc carbohydrate and serum antibodies against it provides the molecular link to cancer: French NutriNet-Santé study
Source: BMC Med. 2020 Sep 23;18:262. doi: 10.1186/s12916-020-01721-8 (PMC7510162; doi:10.1186/s12916-020-01721-8)
Supplement: Supplementary file 1 — Additional file 1: Figure S1. Measurements of anti-Neu5Gc IgG in 120 study cohort by ELISA. Figure S2. Distribution of Neu5Gc intake by food source. Figure S3. Increased levels and diversity of anti-Neu5Gc IgG with higher Neu5Gc intake. Figure S4. Anti-Neu5Gc IgG response in patients with infectious mononucleosis and controls. Figure S5. Characteristics of affinity-purified anti-Neu5Gc antibodies of women 45-60. Figure S6. International cancer risk according to national meat intake. Table S1. Sialic acid content (Neu5Ac and Neu5Gc) in common French food items measured by DMB-HPLC. Table S2. Daily Neu5Gc intake in NutriNet-Santé participants (May 2009 through May 2015) with a minimum of six 24-hour dietary records (total 16,149 participants). Table S3. List of glycans printed on glycan microarray and their characteristics. Table S4. Gcemic index. [file 12916_2020_1721_MOESM1_ESM.pdf]

Additional file 1: Supplementary information

Association between Neu5Gc carbohydrate and serum antibodies against it provide the molecular link to cancer: French NutriNet-Santé study

Salam Bashir[1‡], Leopold K. Fezeu[2‡], Shani Leviatan Ben-Arye[1], Sharon Yehuda[1], Eliran Moshe Reuven[1], Fabien Szabo de Edelenyi[2], Imen Fellah-Hebia[3], Thierry Le Tourneau[4], Berthe Marie Imbert-Marcille[5], Emmanuel B. Drouet[6], Mathilde Touvier[2], Jean-Christian Roussel[3], Hai Yu[7], Xi Chen[7], Serge Hercberg[2], Emanuele Cozzi[8], Jean-Paul Soulillou[9], Pilar Galan[2] and Vered Padler-Karavani[1,*]

[1]Department of Cell Research and Immunology, The George S. Wise Faculty of Life Sciences, Tel Aviv University, Tel Aviv, 69978, Israel; [2]Sorbonne Paris Cité Epidemiology and Statistics Research Center (CRESS), Inserm U1153, Inra U1125, Cnam, Paris 13 University, Nutritional Epidemiology Research Team (EREN), Bobigny, France; [3]Department of Thoracic and Cardiovascular Surgery and [4]Department of Cardiology, Institut du Thorax, University Hospital, Nantes, France; [5]Service de virologie Centre Hospitalo-Universitaire de Nantes, Nantes, F44093, France; [6]Institute of Structural Biology, University Grenoble Alpes, UMR CNRS CEA UGA 5545 CEA, CNRS 38044 Grenoble, F38042, France; [7]Department of Chemistry, University of California-Davis, Davis, CA 95616, USA; [8]Transplant Immunology Unit, Department of Cardiac, Thoracic and Vascular Sciences, Padua University Hospital, Padua, Italy; [9]Centre de Recherche en Transplantation et Immunologie UMR 1064, INSERM, Université de Nantes, Nantes, France.

‡ Contributed equally

***Corresponding author:** Vered Padler-Karavani: Department of Cell Research and Immunology, The George S. Wise Faculty of Life Sciences, Tel Aviv University, Tel Aviv 69978 Israel. Tel: +972-3-640-6737. Fax: +972-3-642-2046. E-mail address: vkaravani@tauex.tau.ac.il

Figure S1.

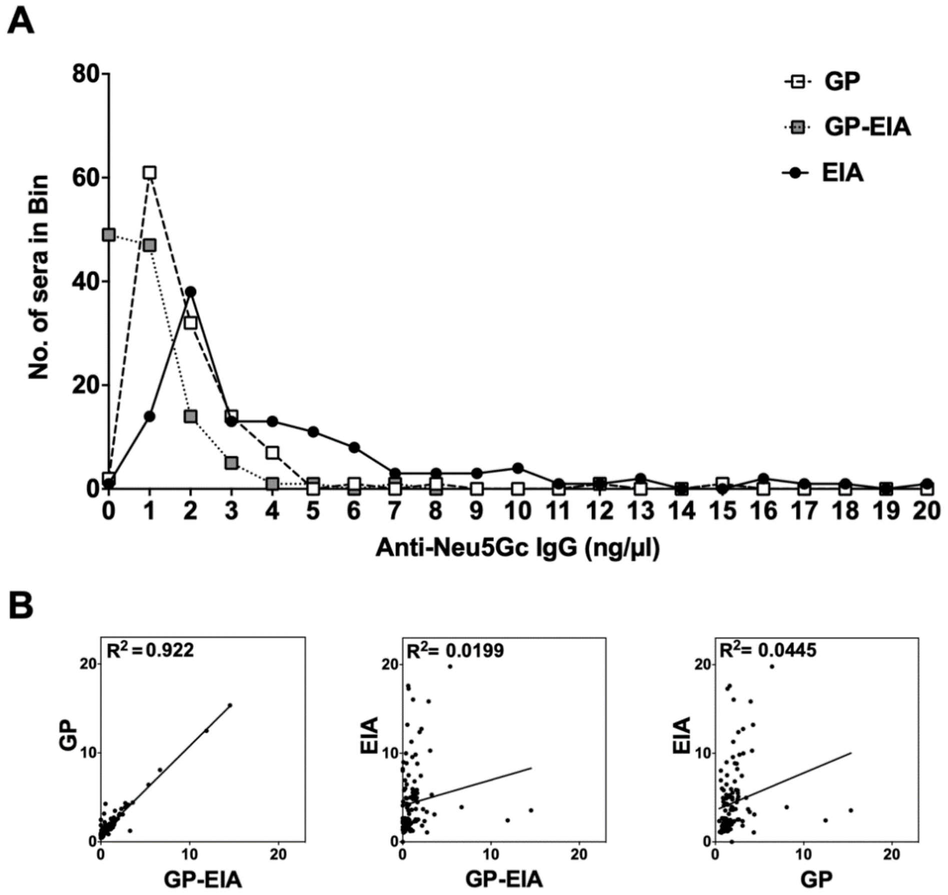

Fig. S1. Measurements of anti-Neu5Gc IgG in 120 study cohort by ELISA. (**A**) Distribution of serum anti-Neu5Gc IgG levels. Anti-Neu5Gc levels were quantified by three different ELISA methods against coated Neu5Gc-glycoproteins (EIA) or Neu5Gc-glycopeptides (GP, GP-EIA), then detected by HRP-anti-human IgG (H+L) (mean ± sem; GP: 2 ± 1.9 ng/µl, GP-EIA: 1.1 ± 1.9 ng/µl, EIA: 4.4 ± 3.9 ng/µl). (**B**) Strong correlation between GP and GP-EIA assays, while no correlation between EIA and GP-EIA or GP assays (linear regression).

Figure S2.

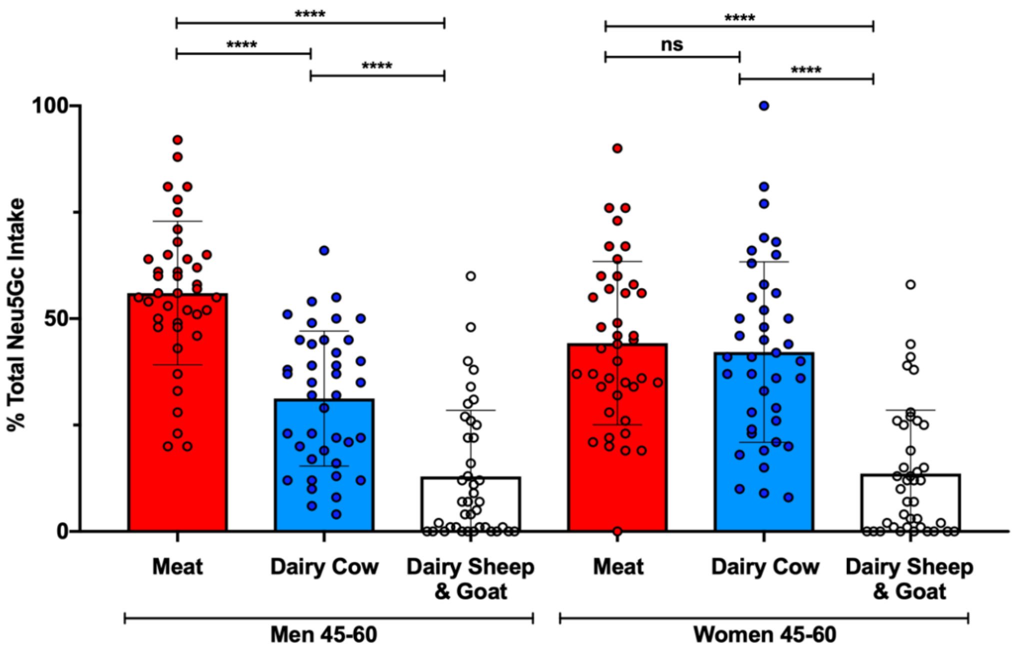

Fig. S2. Distribution of Neu5Gc intake by food source. In the 45-60 age group (40 men and 40 women), daily Neu5Gc intake was calculated per food source and plotted by gender. Each dot represents an individual donor (n=40 per column). Dietary Neu5Gc was dominantly contributed from red meat in men, while dairy in women (One-way ANOVA, with Bonferroni posttest, ****, $p<0.0001$). Total Neu5Gc intake across quartiles in men was 56% ± 2.7% for red meat, while 31% ± 2.5% for dairy cow, and 12.9% ± 2.4% for dairy sheep & goat, and in women, 44.2% ± 3% red meat, 42.1% ± 3.3% dairy cow, 13.6% ± 2.4% dairy sheep & goat (mean ± sem).

Figure S3.

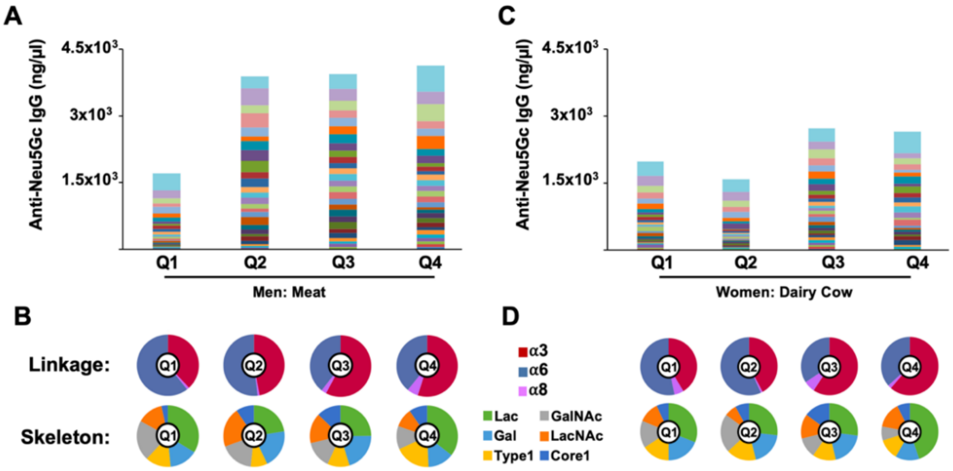

Fig. S3. Increased levels and diversity of anti-Neu5Gc IgG with higher Neu5Gc intake. (**A**) Men 45-60 were stratified according to their Neu5Gc-intake from red meat (Q1-Q4), then the levels and diversity of anti-Neu5Gc IgG reactivity plotted. Each colored-bar represents the sum IgG response per Neu5Gc-glycan across individuals, in each quartile. There is a clear increase in anti-Neu5Gc IgG levels between Q1 and Q2-Q4. (**B**) Pie charts of the sum anti-Neu5Gc IgG response (from **A**) divided according to reactivity against Neu5Gc-glycans with different Sia-linkages (top; α3, α6, α8) or underlying glycans (bottom), per quartile. This shows clear differences in diversity with increased levels of Neu5Gc α3-linkage and Lac (lactose) underlying glycan skeleton with higher Neu5Gc intake. (**C**) Women 45-60 were stratified according to their Neu5Gc-intake from dairy cow (Q1-Q4), then the levels and diversity of anti-Neu5Gc IgG reactivity plotted (as in **A**), demonstrating a clear increase in anti-Neu5Gc IgG levels between Q1/Q2 and Q3-Q4. (**D**) Pie charts of the sum anti-Neu5Gc IgG response (from C) divided by reactivity against Neu5Gc-glycans characteristics, showing clear differences in diversity. There are increased levels of Neu5Gc α3-linkage and Lac (lactose) underlying glycan skeleton with higher Neu5Gc intake.

Figure S4.

A

	IMN	Healthy
N	45	43
Age, in years	24 ± 12	24 ± 12
Men	20 (56%)	23 (53%)
Women	25 (44%)	20 (47%)

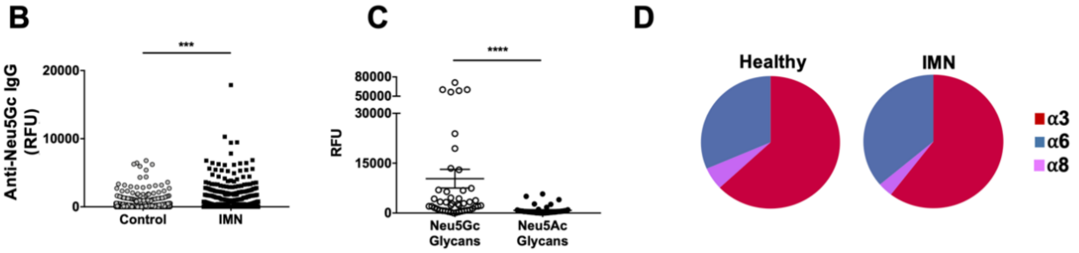

Fig. S4. Anti-Neu5Gc IgG response in patients with infectious mononucleosis and controls. (**A**) Human serum samples were obtained from patients with infectious mononucleosis (IMN) and age/gender matched healthy controls. (**B**) Serum samples (diluted at 1/100) were examined on sialoglycan microarrays printed with 19 Neu5Gc-glycans and their matching 19 Neu5Ac-glycans (glycan IDs 1–10 omitted because they did not pass quality control threshold), then IgG reactivity detected with Cy3-anti-human IgG in relative fluorescent units (RFU), showing higher anti-Neu5Gc IgG reactivity in IMN patients compared to controls (each dot is IgG response per Neu5Gc-glycan per serum; mean ± sem; unpaired Mann–Whitney test; *** p=0.0003). (**C**) IgG reactivity revealed extremely high specificity against Neu5Gc-glycans, with minimal reactivity against Neu5Ac-glycans (each dot represents the sum IgG response against all glycans per serum; Wilcoxon matched paired test, **** p < 0.0001). (**D**) Pie charts of the sum anti-Neu5Gc IgG response divided according to reactivity against Neu5Gc-glycans with different Sia-linkages (α3, α6, α8), showed no difference between the two groups, supporting no altered diversity.

Figure S5.

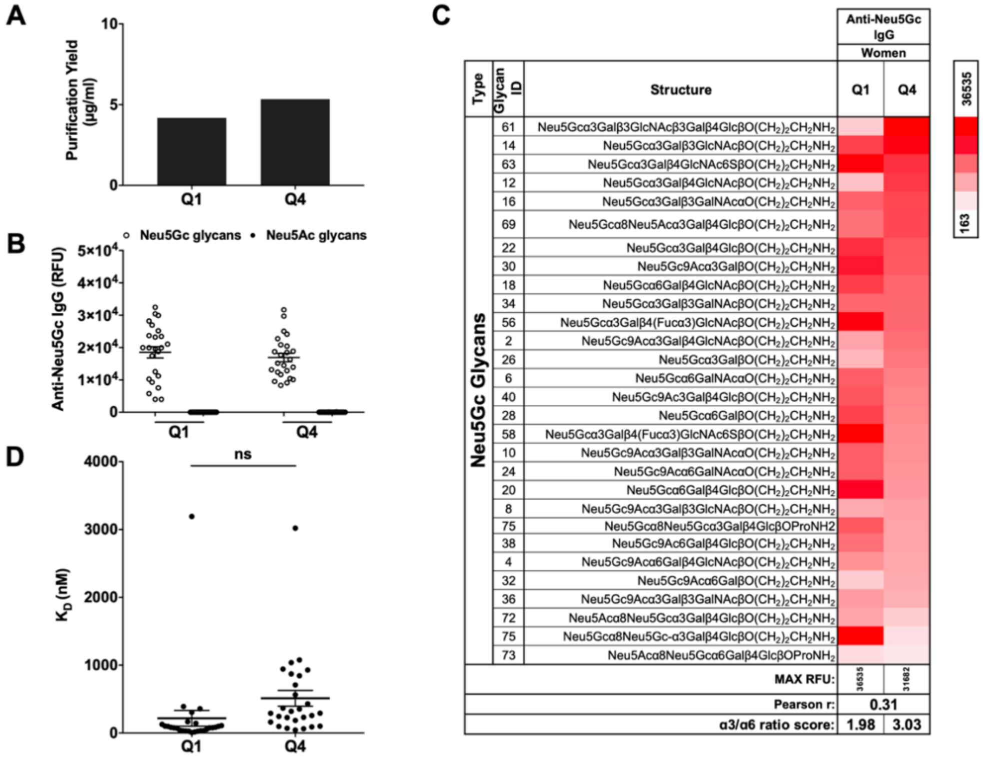

Fig. S5. Characteristics of affinity-purified anti-Neu5Gc antibodies of women 45-60. Anti-Neu5Gc antibodies were affinity-purified from pooled sera of women aged 45-60, with low (Q1) or high (Q4) dairy cow consumption (7.1 ml of Q1, and 7.4 ml of Q4; n=10 per group). (**A**) Total antibody yield was higher in Q4 compared to Q1 (4.18 µg/ml serum versus 5.34 µg/ml serum, respectively). (**B-C**) IgG reactivity examined on sialoglycan microarrays (2 µg/block; detected with Cy3-anti-human IgG) revealed extremely high specificity against Neu5Gc-glycans, and no reactivity against their counterpart Neu5Ac-glycans (**B**; each dot is IgG response per glycan), with a clear change in diversity of glycan recognition in Q4 compared to Q1 (**c**; Pearson r=0.31). In addition, the α3/α6 linkage ratio score were Q1: 1.98 and Q4: 3.03. (**D**) Affinity (K_D) per glycan was calculated from anti-Neu5Gc IgG reactivity examined on sialoglycan microarrays at 16 serial dilutions (40 – 4.9 × 10^{-3} ng/µl; 266.7 – 0.033 nM; non-linear fit with one-site specific binding), showing no change in affinities with higher Neu5Gc intake (mean ± sem; t-test).

Figure S6.

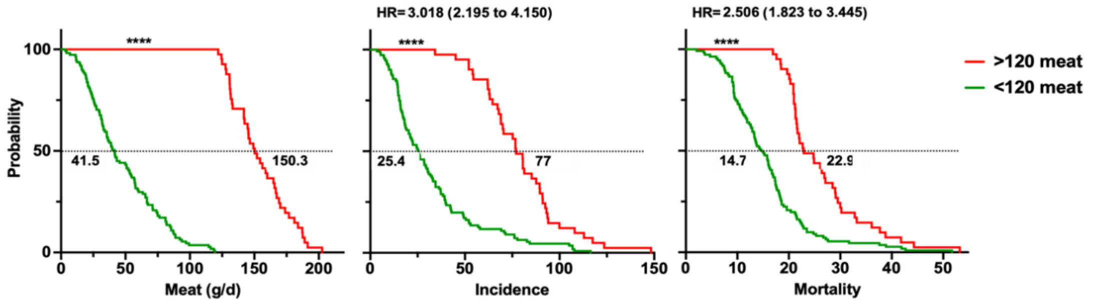

Fig. S6. International cancer risk according to national meat intake. Shows the distribution (probability) of meat consumption, or incidence and mortality rates due to colorectal cancer (age-standardized rates; ASR per 100,000 person-years, including colon, rectum, anus cancers) in different countries comparing two groups by mean intake of meat of above/below 120 gr meat/day. The data is from 152 countries total, 41 countries consumed meat over 120 g/day while 111 countries consumed below 120 g/day. Dividing international meat-cancer risk according to national intake of above/below 120 gr meat shows an increase of 3-fold in incidence and 2.5-fold in mortality in nations that consume >120 gr meat [Survival proportions calculated by Log-rank (Mantel-Cox) test; **** p=0.0001; Median survival marked in dotted lines; Hazard Ratio (HR) and their 95% CI of ratio were calculated by logrank method].

Table S1. Sialic acid content (Neu5Ac and Neu5Gc) in common French food items measured by DMB-HPLC.

Source	Food item	Sialic acid type	
		Neu5Ac	Neu5Gc
		Average ± sem (nmol/gr)	Average ± sem (nmol/gr)
Dairy			
Buffalo	Mozzarella	138 ± 43	6 ± 1
Cow	Parmesan	842 ± 94	11 ± 2
	Crème fraiche	501 ± 126	11 ± 4
	Gouda	562 ± 169	18 ± 6
	Plain yogurt	515 ± 163	18 ± 7
	Petit Suisse	461 ± 222	18 ± 9
	Powdered milk	1847 ± 279	107 ± 36
	Milk	781 ± 101	21 ± 4
	Cheese strainer	557 ± 141	21 ± 8
	Fromage blanc	549 ± 91	23 ± 11
	Cheese spread	664 ± 145	23 ± 13
	Camembert	987 ± 144	59 ± 7
Goat	Feta	239 ± 11	280 ± 15
	Soft goat cheese	278 ± 49	336 ± 62
	Roll of goat cheese	431 ± 120	344 ± 104
	Dry cheese	399 ± 138	533 ± 211

**Sheep**	**Yogurt**	153 ± 46	277 ± 85
	**Roquefort**	49 ± 2	550 ± 13
	**Etorki**	81 ± 8	633 ± 78
**Red and processed meat**			
**Cow**	**Dried sausage**	292 ± 60	22 ± 9
	**Chipolatas sausages**	395 ± 252	32 ± 3
	**Inards**	418 ± 142	110 ± 65
	**Beef tongue**	257 ± 123	131 ± 124
	**Beef**	234 ± 33	163 ± 17
	**Pate**	196 ± 85	307 ±240
**Pig**	**Pork**	90 ± 12	18 ± 3
	**Bacon strips**	368 ± 215	31 ± 15
	**Pork rillettes**	122 ± 21	68 ± 41
	**Ham**	349 ± 239	71 ± 48
	**Cured ham**	232 ± 49	140 ± 89
	**Strasbourg sausage**	121 ± 72	207 ± 174
	**Liver**	473 ± 107	409 ± 63
**Lamb**		324 ± 224	67 ± 58
**Rabbit**		164 ± 73	7 ± 4

Table S2. Daily Neu5Gc intake in NutriNet-Santé participants (May 2009 through May 2015) with a minimum of six 24-hour dietary records (total 16,149 participants).

**Group**	**N**	**Daily Neu5Gc intake (µmol/day) (calculated from ≥ 6 24h-dietary records)**			
		**Min**	**Mean**	**Median**	**Max**
**By quartile**					
**Q1**	4037	0	8.2	8.9	11.5
**Q2**	4037	11.5	13.6	13.7	15.7
**Q3**	4038	15.7	18	17.9	20.9
**Q4**	4037	20.9	28.4	25.6	181.5
**By gender**					
**Men**	4562	0	19.7	18.4	167.6
**Women**	11587	0	16.1	14.8	181.5
**By age and gender (Men)**					
**18-30 y**	216	0	17.6	16.5	66.8
**31-44 y**	588	0	18	16.6	85.9
**45-60 y**	1240	0.1	20	18.9	83.2
**> 60 y**	2518	0	20.1	18.7	167.6
**By age and gender (Women)**					
**18-30 y**	1006	0	13.6	13.1	64
**31-44 y**	2040	0	15	14	93.3
**45-60 y**	4779	0	16.2	14.9	104.7
**> 60 y**	3762	0	17.1	15.6	181.5

Table S3. List of glycans printed on glycan microarray and their characteristics. Sia-linkages (Siaα2–3/6/8 linkages; α3, α6, α8, respectively) or underlying skeleton glycans [Lac (lactose; Galβ4Glc), Gal (galactose), Type 1 (Galβ3GlcNAc), GalNAc (*N*-acetylgalactoseamine), LacNAc (*N*-acetyllactoseamine; Galβ4GlcNAc), Core 1 (Galβ3GalNAc)].

Glycan ID	Structure	Sialic Acid		Skeleton	Pairs of Neu5Gc/ Neu5Ac glycans
		Type	Linkage		
1	Neu5,9Ac$_2$α3Galβ4GlcNAcβO(CH$_2$)$_3$NH$_2$	Neu5,9Ac$_2$	α3	LacNAc	P1-Ac
2	Neu5Gc9Acα3Galβ4GlcNAcβO(CH$_2$)$_3$NH$_2$	Neu5Gc9Ac	α3	LacNAc	P1-Gc
3	Neu5,9Ac$_2$α6Galβ4GlcNAcβO(CH$_2$)$_3$NH$_2$	Neu5,9Ac$_2$	α6	LacNAc	P2-Ac
4	Neu5Gc9Acα6Galβ4GlcNAcβO(CH$_2$)$_3$NH$_2$	Neu5Gc9Ac	α6	LacNAc	P2-Gc
5	Neu5Acα6GalNAcαO(CH$_2$)$_3$NH$_2$	Neu5Ac	α6	GalNAc	P3-Ac
6	Neu5Gcα6GalNAcαO(CH$_2$)$_3$NH$_2$	Neu5Gc	α6	GalNAc	P3-Gc
7	Neu5,9Ac$_2$α3Galβ3GlcNAcβO(CH$_2$)$_3$NH$_2$	Neu5,9Ac$_2$	α3	Type 1	P4-Ac
8	Neu5Gc9Acα3Galβ3GlcNAcβO(CH$_2$)$_3$NH$_2$	Neu5Gc9Ac	α3	Type 1	P4-Gc
9	Neu5,9Ac$_2$α3Galβ3GalNAcαO(CH$_2$)$_3$NH$_2$	Neu5,9Ac$_2$	α3	Core 1	P5-Ac
10	Neu5Gc9Acα3Galβ3GalNAcαO(CH$_2$)$_3$NH$_2$	Neu5Gc9Ac	α3	Core 1	P5-Gc
11	Neu5Acα3Galβ4GlcNAcβO(CH$_2$)$_3$NH$_2$	Neu5Ac	α3	LacNAc	P6-Ac
12	Neu5Gcα3Galβ4GlcNAcβO(CH$_2$)$_3$NH$_2$	Neu5Gc	α3	LacNAc	P6-Gc
13	Neu5Acα3Galβ3GlcNAcβO(CH$_2$)$_3$NH$_2$	Neu5Ac	α3	Type 1	P7-Ac
14	Neu5Gcα3Galβ3GlcNAcβO(CH$_2$)$_3$NH$_2$	Neu5Gc	α3	Type 1	P7-Gc
15	Neu5Acα3Galβ3GalNAcαO(CH$_2$)$_3$NH$_2$	Neu5Ac	α3	Core 1	P8-Ac
16	Neu5Gcα3Galβ3GalNAcαO(CH$_2$)$_3$NH$_2$	Neu5Gc	α3	Core 1	P8-Gc
17	Neu5Acα6Galβ4GlcNAcβO(CH$_2$)$_3$NH$_2$	Neu5Ac	α6	LacNAc	P9-Ac
18	Neu5Gcα6Galβ4GlcNAcβO(CH$_2$)$_3$NH$_2$	Neu5Gc	α6	LacNAc	P9-Gc
19	Neu5Acα6Galβ4GlcβO(CH$_2$)$_3$NH$_2$	Neu5Ac	α6	Lactose	P10-Ac
20	Neu5Gcα6Galβ4GlcβO(CH$_2$)$_3$NH$_2$	Neu5Gc	α6	Lactose	P10-Gc
21	Neu5Acα3Galβ4GlcβO(CH$_2$)$_3$NH$_2$	Neu5Ac	α3	Lactose	P11-Ac
22	Neu5Gcα3Galβ4GlcβO(CH$_2$)$_3$NH$_2$	Neu5Gc	α3	Lactose	P11-Gc
23	Neu5,9Ac$_2$α6GalNAcαO(CH$_2$)$_3$NH$_2$	Neu5,9Ac$_2$	α6	GalNAc	P12-Ac
24	Neu5Gc9Acα6GalNAcαO(CH$_2$)$_3$NH$_2$	Neu5Gc9Ac	α6	GalNAc	P12-Gc
25	Neu5Acα3GalβO(CH$_2$)$_3$NH$_2$	Neu5Ac	α3	Galactose	P13-Ac
26	Neu5Gcα3GalβO(CH$_2$)$_3$NH$_2$	Neu5Gc	α3	Galactose	P13-Gc
27	Neu5Acα6GalβO(CH$_2$)$_3$NH$_2$	Neu5Ac	α6	Galactose	P14-Ac
28	Neu5Gcα6GalβO(CH$_2$)$_3$NH$_2$	Neu5Gc	α6	Galactose	P14-Ac

29	Neu5,9Ac$_2$α3GalβO(CH$_2$)$_3$NH$_2$	Neu5,9Ac$_2$	α3	Galactose	P15-Gc
30	Neu5Gc9Acα3GalβO(CH$_2$)$_3$NH$_2$	Neu5Gc9Ac	α3	Galactose	P15-Ac
31	Neu5,9Ac$_2$α6GalβO(CH$_2$)$_3$NH$_2$	Neu5,9Ac$_2$	α6	Galactose	P16-Gc
32	Neu5Gc9Acα6GalβO(CH$_2$)$_3$NH$_2$	Neu5Gc9Ac	α6	Galactose	P16-Ac
33	Neu5Acα3Galβ3GalNAcβO(CH$_2$)$_3$NH$_2$	Neu5Ac	α3	Core 1	P17-Gc
34	Neu5Gcα3Galβ3GalNAcβO(CH$_2$)$_3$NH$_2$	Neu5Gc	α3	Core 1	P17-Ac
35	Neu5,9Ac$_2$α3Galβ3GalNAcβO(CH$_2$)$_3$NH$_2$	Neu5,9Ac$_2$	α3	Core 1	P18-Gc
36	Neu5Gc9Acα3Galβ3GalNAcβO(CH$_2$)$_3$NH$_2$	Neu5Gc9Ac	α3	Core 1	P18-Ac
37	Neu5,9Ac$_2$α6Galβ4GlcβO(CH$_2$)$_3$NH$_2$	Neu5,9Ac$_2$	α6	Lactose	P19-Gc
38	Neu5Gc9Ac6Galβ4GlcβO(CH$_2$)$_3$NH$_2$	Neu5Gc9Ac	α6	Lactose	P19-Ac
39	Neu5,9Ac$_2$α3Galβ4GlcβO(CH$_2$)$_3$NH$_2$	Neu5,9Ac$_2$	α3	Lactose	P20-Ac
40	Neu5Gc9Ac3Galβ4GlcβO(CH$_2$)$_3$NH$_2$	Neu5Gc9Ac	α3	Lactose	P20-Gc
41	Neu5Acα8Neu5Acα3Galβ4GlcβO(CH$_2$)$_3$NH$_2$	Neu5Ac-Neu5Ac	α3-α8	Lactose	
42	Neu5Acα8Neu5Acα8Neu5Acα3Galβ4GlcβO(CH$_2$)$_3$NH$_2$	(Neu5Ac)$_3$	α3-α8	Lactose	
55	Neu5Acα3Galβ4(Fucα3)GlcNAcβO(CH$_2$)$_3$NH$_2$	Neu5Ac	α3	Le^x	P21-Ac
56	Neu5Gcα3Galβ4(Fucα3)GlcNAcβO(CH$_2$)$_3$NH$_2$	Neu5Gc	α3	Le^x	P21-Gc
57	Neu5Acα3Galβ4(Fucα3)GlcNAc6SβO(CH$_2$)$_3$NH$_2$	Neu5Ac	α3	6S-Le^x	P22-Ac
58	Neu5Gcα3Galβ4(Fucα3)GlcNAc6SβO(CH$_2$)$_3$NH$_2$	Neu5Gc	α3	6S-Le^x	P22-Gc
60	Neu5Acα3Galβ3GlcNAcβ3Galβ4GlcβO(CH$_2$)$_3$NH$_2$	Neu5Ac	α3	LNT	P23-Ac
61	Neu5Gcα3Galβ3GlcNAcβ3Galβ4GlcβO(CH$_2$)$_3$NH$_2$	Neu5Gc	α3	LNT	P23-Gc
62	Neu5Acα3Galβ4GlcNAc6SβO(CH$_2$)$_3$NH$_2$	Neu5Ac	α3	6S-LacNAc	P24-Ac
63	Neu5Gcα3Galβ4GlcNAc6SβO(CH$_2$)$_3$NH$_2$	Neu5Gc	α3	6S-LacNAc	P24-Gc
67	Neu5Acα6(Neu5Gcα3)Galβ4GlcβO(CH$_2$)$_3$NH$_2$	Neu5Ac/Neu5Gc	α3/α6	Lactose	
69	Neu5Gcα8Neu5Acα3Galβ4GlcβO(CH$_2$)$_3$NH$_2$	Neu5Gc-Neu5Ac	α3-α8	Lactose	
72	Neu5Acα8Neu5Gcα3Galβ4GlcβO(CH$_2$)$_3$NH$_2$	Neu5Ac-Neu5Gc	α3-α8	Lactose	
73	Neu5Acα8Neu5Gcα6Galβ4GlcβO(CH$_2$)$_3$NH$_2$	Neu5Ac-Neu5Gc	α6-α8	Lactose	
75	Neu5Gcα8Neu5Gcα3Galβ4GlcβO(CH$_2$)$_3$NH$_2$	Neu5Gc-Neu5Gc	α3-α8	Lactose	
78	Galα3Galβ4GlcNAcβO(CH$_2$)$_3$NH$_2$	N/A	N/A	LacNAc	αGal

Table S4. Gcemic index. Gram of food to consume to reach daily nmol Neu5Gc per quartile based on Neu5Gc content (measured by DMB-HPLC). Range minimum is the average daily Neu5Gc consumption by women per quartile (Q1 min women 6937 nmol/day; Q4 min women 19627 nmol/day), and range maximum is the average Neu5Gc consumption by men per quartile (Q1 max men 9443 nmol/day; Q4 max men 25265 nmol/day). *Gcemic index* is the Neu5Gc content (nmol/gr) in each food item relative to the amount measured in beef (163 nmol/gr). *Inverse Gcemic index* (1/ *Gcemic index*) is the fold change in gr food to consume to reach the Neu5Gc quantities as in beef (Mozzarella has a Gcemic index of 0.03 hence 28.9 times more grams can be consumed compared to beef).

Source		Food item	Neu5Gc Average ± sem (nmol/gr)	Q1 Range Min − Max (gr)	Q4 Range Min − Max (gr)	Gcemic index	Inverse Gcemic index
Dairy	Buffalo	Mozzarella	6 ± 1	1223 − 1664	3459 − 4453	0.03	28.90
Meat	Rabbit	Rabbit	7 ± 4	962 − 1310	2723 − 3505	0.04	22.75
Dairy	Cow	Crème fraiche	11 ± 4	623 − 848	1762 − 2268	0.07	14.72
Dairy	Cow	Parmesan	11 ± 2	604 − 822	1708 − 2198	0.07	14.27
Meat	Pig	Pork	18 ± 3	393 − 536	1113 − 1433	0.11	9.30
Dairy	Cow	Plain yogurt	18 ± 7	382 − 519	1079 − 1390	0.11	9.02
Dairy	Cow	Petit Suisse	18 ± 9	378 − 514	1069 − 1375	0.11	8.93
Dairy	Cow	Gouda	18 ± 6	377 − 514	1068 − 1375	0.11	8.92
Dairy	Cow	Milk	21 ± 4	332 − 452	939 − 1209	0.13	7.84
Dairy	Cow	Cheese strainer	21 ± 8	325 − 443	920 − 1184	0.13	7.68
Meat	Cow	Dried sausage	22 ± 9	318 − 433	900 − 1159	0.13	7.52
Dairy	Cow	Fromage blanc	23 ± 11	300 − 409	850 − 1094	0.14	7.10
Dairy	Cow	Cheese spread	23 ± 13	300 − 408	848 − 1092	0.14	7.08
Meat	Pig	Bacon strips	31 ± 15	224 − 304	633 − 814	0.19	5.28
Meat	Cow	Sausages	32 ± 3	219 − 298	619 − 796	0.19	5.17
Dairy	Cow	Camembert	59 ± 7	118 − 161	335 − 431	0.36	2.80
Meat	Lamb	Lamb	67 ± 58	104 − 141	294 − 378	0.41	2.46
Meat	Pig	Pork rillettes	68 ± 41	102 − 139	289 − 372	0.41	2.41
Meat	Pig	Ham	71 ± 48	98 − 133	276 − 355	0.43	2.31

Dairy	Cow	Powdered milk	107 ± 36	65 − 88	183 − 236	0.65	1.53
Meat	Cow	Inards	110 ± 65	63 − 86	178 − 229	0.67	1.49
Meat	Cow	Beef tongue	131 ± 124	53 − 72	150 − 193	0.80	1.25
Meat	Pig	Cured ham	140 ± 89	50 − 67	140 − 181	0.85	1.17
**Meat**	**Cow**	**Beef**	**163 ± 17**	**23 − 58**	**120 − 154**	**1.00**	**1.00**
Meat	Pig	Sausage	207 ± 174	34 − 46	95 − 122	1.26	0.79
Dairy	Sheep	Yogurt	277 ± 85	25 − 34	71 − 91	1.69	0.59
Dairy	Goat	Feta	280 ± 15	25 − 34	70 − 90	1.71	0.59
Meat	Cow	Pate	307 ±240	23 − 31	64 − 82	1.87	0.53
Dairy	Goat	Soft goat cheese	336 ± 62	21 − 28	58 − 75	2.05	0.49
Dairy	Goat	Roll of cheese	344 ± 104	20 − 27	57 − 73	2.10	0.48
Meat	Pig	Liver	409 ± 63	17 − 23	48 − 62	2.50	0.40
Dairy	Goat	Dry cheese	533 ± 211	13 − 18	37 − 47	3.25	0.31
Dairy	Sheep	Roquefort	550 ± 13	13 − 17	36 − 46	3.35	0.30
Dairy	Sheep	Etorki	633 ± 78	11 − 15	31 − 40	3.86	0.26
